# Changes in *Medicago truncatula* seed proteome along the rehydration–dehydration cycle highlight new players in the genotoxic stress response

**DOI:** 10.3389/fpls.2023.1188546

**Published:** 2023-06-13

**Authors:** Andrea Pagano, Laura Kunz, Antje Dittmann, Susana De Sousa Araújo, Anca Macovei, Shraddha Shridhar Gaonkar, Federico Sincinelli, Hisham Wazeer, Alma Balestrazzi

**Affiliations:** ^1^ Department of Biology and Biotechnology ‘L. Spallanzani’, University of Pavia, Pavia, Italy; ^2^ Functional Genomics Center Zurich (FGCZ), University of Zurich/Eidgenossische Technische Hochschule (ETH) Zurich, Zurich, Switzerland; ^3^ Association BLC3 - Campus of Technology and Innovation, Centre BIO R&D Unit | North Delegation, Macedo de Cavaleiros, Portugal; ^4^ National Biodiversity Future Center (NBFC), Palermo, Italy

**Keywords:** hydropriming, *Medicago truncatula*, seed proteome, inosine triphosphate pyrophosphorylase, 2’-deoxyinosine, comet assay

## Abstract

**Introduction:**

Several molecular aspects underlying the seed response to priming and the resulting vigor profile are still poorly understood. Mechanisms involved in genome maintenance deserve attention since the balance between stimulation of germination and DNA damage accumulation versus active repair is a key determinant for designing successful seed priming protocols.

**Methods:**

Changes in the Medicago truncatula seed proteome were investigated in this study, using discovery mass spectrometry and label-free quantification, along the rehydration-dehydration cycle of a standard vigorization treatment (hydropriming plus dry-back), and during post-priming imbibition.

**Resuts and discussion:**

From 2056 to 2190 proteins were detected in each pairwise comparison, among which six were differentially accumulated and 36 were detected only in one condition. The following proteins were selected for further investigation: MtDRP2B (DYNAMIN-RELATED PROTEIN), MtTRXm4 (THIOREDOXIN m4), and MtASPG1 (ASPARTIC PROTEASE IN GUARD CELL 1) showing changes in seeds under dehydration stress; MtITPA (INOSINE TRIPHOSPHATE PYROPHOSPHORYLASE), MtABA2 (ABSCISIC ACID DEFICIENT 2), MtRS2Z32 (SERINE/ARGININE-RICH SPLICING FACTOR RS2Z32), and MtAQR (RNA HELICASE AQUARIUS) that were differentially regulated during post-priming imbibition. Changes in the corresponding transcript levels were assessed by qRT-PCR. In animal cells, ITPA hydrolyses 2’-deoxyinosine triphosphate and other inosine nucleotides, preventing genotoxic damage. A proof of concept was performed by imbibing primed and control M. truncatula seeds in presence/absence of 20 mM 2’-deoxyinosine (dI). Results from comet assay highlighted the ability of primed seeds to cope with dI-induced genotoxic damage. The seed repair response was assessed by monitoring the expression profiles of MtAAG (ALKYL-ADENINE DNA GLYCOSILASE) and MtEndoV (ENDONUCLEASE V) genes that participate in the repair of the mismatched I:T pair in BER (base excision repair) and AER (alternative excision repair) pathways, respectively.

## Introduction

The current challenges of climate change and pandemic crises require highly effective strategies addressing the agrifood chain at all levels, in order to ensure climate-ready crops capable to perform under adverse environments (FAO, 2021; Razzaq et al., 2021; Xiong et al., 2022). In this scenario, seed enhancement technologies have gained attention. High-quality seeds, proxy of increased crop yields, are obtained with low-cost, pre-sowing treatments (seed priming) that improve germination (Heydecker et al., 1973; Paparella et al., 2015; Farooq et al., 2019; Fabrissin et al., 2021; Reed et al., 2022; Pagano et al., 2023). During the priming treatment, imbibition is carried out under controlled conditions to boost the seed antioxidant and DNA repair responses, key components of the pre-germinative metabolism (Bailly, 2004; Macovei et al., 2017). Seeds are brought towards a more advanced physiological stage; however, the treatment must stop before radicle protrusion to avoid loss of desiccation tolerance (Soeda et al., 2005). At the moment, the empirical features of priming protocols and their genotype- and seed lot-dependent variability delay the work of seed technologists, breeders, and seed bank operators. Molecular hallmarks (genes, proteins, and metabolites) are required to predict and monitor the effectiveness of novel, tailored pre-sowing treatments (Paparella et al., 2015; Macovei et al., 2017; Farooq et al., 2019; Fabrissin et al., 2021). In order to address such a need, the existing gap of knowledge on the molecular mechanisms underlying the seed response to priming should be shortened.

Seed vigor is a complex trait resulting from multiple levels of regulation and interactions, in response to both genetic and environmental factors. Issues related to seed vigor can be investigated using high-throughput techniques, among which is proteomics that provides a comprehensive picture of the cellular metabolism at the protein level (Smolikova et al., 2020; Wang et al., 2022). The seed proteome and its dynamic responses to environmental stresses have been explored in the attempt to find out the key proteins associated with seed vigor (Catusse et al., 2011; Yacoubi et al., 2011; Rajjou et al., 2012; Kubala et al., 2015; Wang et al., 2015a; Zhang et al., 2015). The impact of priming on the seed proteome in relation to improved stress resilience has been reported for different species, e.g., ascorbate-primed wheat (*Triticum* spp.) seeds under salt stress (Fercha et al., 2014), melatonin-primed corn (*Zea mays* L.) seeds exposed to chilling (Kołodziejczyk et al., 2016), and β-aminobutyric acid-treated barley (*Hordeum vulgare* L.) seeds under salt stress (Mostek et al., 2016). Seed proteome analysis has allowed the identification of proteins useful to support selective breeding, as in the case of elite barley accessions used in the malt industry (Osama et al., 2021). The proteomic profiles of aged and melatonin-primed oat (*Avena sativa* L.) seeds revealed that improved antioxidant capacity and restoration of membrane integrity were essential to promote germination performance (Yan and Mao, 2021). The impact of hydro-electro hybrid priming applied to carrot (*Daucus carota* L.) seeds was explored through proteomics, highlighting the concerted regulation of protein synthesis and degradation, and respiratory metabolism (Zhao et al., 2022).

Despite these extensive studies, several molecular aspects underlying the seed response to priming and the resulting vigor profile are still poorly understood (Smolikova et al., 2020; Pagano et al., 2023). Mechanisms involved in genome maintenance deserve attention since the balance between stimulation of germination and DNA damage accumulation *versus* active repair is a key determinant for designing successful priming protocols (Pagano et al., 2023). The combined effect of enhanced antioxidant activity and DNA repair contributes to preserve genome integrity during early seed imbibition (Bailly, 2004; Macovei et al., 2010; Balestrazzi et al., 2011; Macovei et al., 2011; Pagano et al., 2017; Pagano et al., 2019; Waterworth et al., 2019; Waterworth et al., 2022; Pagano et al. 2022a; 2022b; Forti et al., 2020a; Forti et al., 2020b; Pagano et al., 2023).

To our knowledge, proteomics insights into the DNA damage response at the seed level are still missing. The analysis of changes in the abundance of proteins involved in genotoxic stress tolerance might add novel information useful for both basic and applied research purposes. As reported for animal cells, proteomic studies provide a global picture of the cell response to specific DNA damage inducers, allowing a better understanding of the underlying mechanisms (von Stechow and Olsen, 2017). However, the potential of this high-throughput approach can be further exploited by using the resulting data as a source of novel biomarkers correlated with a specific network of events (protein signature) (von Stechow and Olsen, 2017).

In this study, a label-free LC-MS/MS proteomics approach was used to characterize the *Medicago truncatula* seed proteome along the rehydration/dehydration cycle of a standard vigorization treatment [hydropriming (HP) plus dry-back] and during post-priming imbibition to tackle changes correlated to improved germination performance and identify novel players involved in the genotoxic stress response in seeds. Considering the impact of genotoxic stress on the seed response to priming and the related open questions (Pagano et al. 2022a; 2022b; Pagano et al., 2023), attention was focused on those proteins with potential or documented roles in genome maintenance, such as MtITPA (INOSINE TRIPHOSPHATE PYROPHOSPHORYLASE) (Yoneshima et al., 2016; Straube et al., 2022). MtITPA was further investigated in the context of seed priming, and its contribution to the enhanced genotoxic stress response of the *M. truncatula* primed seeds is discussed.

## Materials and methods

### Plant material, hydropriming, 2’-deoxy-inosine treatments, and germination tests

*M. truncatula* seeds (commercial genotype, kindly provided by Fertiprado L.d.a., Portugal) were treated as follows. For HP, seeds were transferred to Petri dishes (diameter, 90 mm) containing two filter papers moistened with 2.5 ml of H_2_O, sealed and kept in a growth chamber at 22°C under light conditions with a photon flux density of 150 μmol m^−2^ s^−1^, a photoperiod of 16/8 h, and 70%–80% relative humidity. HP was performed by imbibing seeds for 2 h (Araujo et al., 2019). For each treatment, three independent replications with 20 seeds per replication were analyzed. For dehydration (dry-back, DB), primed seeds were transferred into glass tubes, placed between two cotton disks, covered with silica beads (disidry^®^ Orange Silica Gel, The Aerodyne, Florence, Italy) with a seed:silica ratio of 1:10, and kept at 24–25°C. Seed fresh weight was monitored every 15 min for 6 h, until the dry weight was reached. For germination tests, seeds were transferred to Petri dishes (diameter, 90 mm) containing two filter papers moistened with 2.5 ml of H_2_O, sealed and kept in a growth chamber as previously described. Seeds with protrusion of the primary radicle were considered germinated. For treatments with 2’-deoxyinosine (dI, Sigma-Aldrich, Milan, Italy), unprimed (UP) and 2 h-hydroprimed (P) *M. truncatula* seeds were sown over filter paper in the presence/absence of 2.5 ml of a solution containing 20 mM dI, resulting in four treatments: UP − dI (untreated unprimed seeds), UP + dI (unprimed seeds exposed to dI), P − dI (untreated primed seeds), and P + dI (primed seeds exposed to dI). Germination tests were carried out as previously described. Germination parameters were calculated according to Ranal and Garcia de Santana (2006). Phenotyping was performed by measuring the seedling fresh and dry weight, and radicle length. Samples were collected at the indicated time points, frozen using liquid N_2_, and stored at −80°C for subsequent molecular analyses.

### Sample preparation for LC-MS/MS

For each condition, five replicates (approximately 100 seeds/each) were obtained and ground in the presence of liquid nitrogen. From each replicate, 30–50 mg of seed powder was resuspended in 300 µl of lysis buffer [4% sodium dodecyl sulfate (SDS), Sigma-Aldrich] in 4% SDS/50 mM TEAB (triethylammonium bicarbonate, Sigma-Aldrich)/100 mM DTT (dithiothreitol, Sigma-Aldrich). Protein extraction was carried out using a tissue homogenizer (TissueLyser II, QIAGEN) by applying 2×2 min cycles at 30 Hz, and treating with High-Intensity Focused Ultrasound (HIFU) (1 min at 80% ultrasonic amplitude) followed by boiling at 95°C for10 min under shaking (800 r.p.m.). Protein concentration was estimated using the Qubit^®^ Protein Assay Kit (Life Technologies, Zurich, Switzerland). For each sample, 20 µg of protein was taken and alkylated with 15 mM iodoacetamide for 30 min. Samples were further processed using the single‐pot solid‐phase enhanced sample preparation (SP3). The SP3 protein purification, digest, and peptide clean-up were performed using a KingFisher Flex System (Thermo Fisher Scientific) and Carboxylate-Modified Magnetic Particles (GE Life Sciences; GE65152105050250, GE45152105050250). Beads were conditioned following the manufacturer’s instructions, consisting of three washes with water at a concentration of 1 µg/µl. Samples were diluted with 100% ethanol to a final concentration of 50% ethanol. The beads, wash solutions, and samples were loaded into 96 deep well or micro-plates and transferred to the KingFisher. The following steps were carried out on the robot: collection of beads from the last wash, protein binding to beads, washing of beads in wash solutions 1–3 (80% ethanol), protein digestion (overnight at 37°C with a trypsin:protein ratio of 1:50 in 50 mM TEAB), and peptide elution from the magnetic beads using MilliQ water. The digest solution and water elution were combined and peptides were acidified to perform a stage-tip cleanup using two Empore reversed-phase extraction disks (3M). The eluted samples were dried to completeness and re-solubilized in 20 µl of MS sample buffer (3% acetonitrile, 0.1% formic acid).

### LC-MS/MS data acquisition

LC-MS/MS analysis was performed on an Orbitrap Fusion Lumos (Thermo Scientific) equipped with a Digital PicoView source (New Objective) and coupled to an M-Class UPLC (Waters). Solvent composition of the two channels was 0.1% formic acid for channel A and 99.9% acetonitrile in 0.1% formic acid for channel B. Column temperature was 50°C. For each sample, 1 µl of peptides was loaded on a commercial ACQUITY UPLC M-Class Symmetry C18 Trap Column (100 Å, 5 µm, 180 µm × 20 mm, Waters) connected to an ACQUITY UPLC M-Class HSS T3 Column (100 Å, 1.8 µm, 75 µm × 250 mm, Waters). The peptides were eluted at a flow rate of 300 nl/min. After a 3-min initial hold at 5% B, a gradient from 5% to 22% B in 80 min and 22% to 32% B in an additional 10 min was applied. The column was cleaned after the run by increasing to 95% B and holding 95% B for 10 min prior to re-establishing loading condition. Samples were measured in randomized order. The mass spectrometer was operated in data-dependent mode (DDA) with a maximum cycle time of 3 s, using Xcalibur (tune version 3.1.2412.25), with a spray voltage set to 2.5 kV, funnel RF level at 40%, heated capillary temperature at 275°C, and Advanced Peak Determination (APD) on. Full-scan MS spectra (300–1,500 m/z) were acquired at a resolution of 120,000 at 200 m/z after accumulation to an automated gain control (AGC) target value of 500,000 or for a maximum injection time of 40 ms. Precursors with an intensity above 5,000 were selected for MS/MS. Ions were isolated using a quadrupole mass filter with 1.2 m/z isolation window and fragmented by higher-energy collisional dissociation (HCD) using a normalized collision energy of 35%. Fragments were detected in the linear ion trap with the scan rate set to rapid, the automatic gain control set to 10,000 ions, and the maximum injection time set to 50 ms. Charge state screening was enabled, and singly, unassigned charge states and charge states higher than seven were excluded. Precursor masses previously selected for MS/MS measurement were excluded from further selection for 20 s, applying a mass tolerance of 10 ppm. The samples were acquired using internal lock mass calibration on m/z 371.1012 and 445.1200. The mass spectrometry proteomics data were handled using the local laboratory information management system (LIMS) and all relevant data have been deposited to the ProteomeXchange Consortium *via* the PRIDE (http://www.ebi.ac.uk/pride) partner repository with the dataset identifier PXD031336.

### Peptide identification and quantification

The acquired raw MS data were processed by MaxQuant (version 1.6.2.3), followed by protein identification using the integrated Andromeda search engine. Spectra were searched against an *M. truncatula* database from the UniProt reference proteome (taxonomy 2051, canonical version from 2020-10-02), concatenated to its reversed decoyed fasta database and common protein contaminants. Carbamidomethylation of cysteine was set as fixed modification, while methionine oxidation and N-terminal protein acetylation were set as variable. Enzyme specificity was set to trypsin/P allowing a minimal peptide length of seven amino acids and a maximum of two missed cleavages. MaxQuant IonTrap default search settings were used. The maximum false discovery rate (FDR) was set to 0.01 for peptides and 0.05 for proteins. Label-free quantification was enabled and a 2-min window for match between runs was applied. In the MaxQuant experimental design template, each file is kept separate in the experimental design to obtain individual quantitative values. Protein fold changes were computed based on Intensity values reported in the proteinGroups.txt file. A set of functions implemented in the R package SRM Service was used to filter for proteins with two or more peptides allowing for a maximum of four missing values per comparison, and to normalize the data with a modified robust z-score transformation and to compute *p*-values using the *t*-test with pooled variance. If all measurements of a protein are missing in one of the conditions, a pseudo fold change was computed replacing the missing group average by the mean of 10% smallest protein intensities in that condition.

### Protein and pathway enrichment analysis

Gene set enrichment analysis was carried out in R (v 4.0.2) using the package fgsea (v 1.14.0) and visualized using ggplot2 (v 3.3.3). Proteins were ranked based on their group comparison *t*-statistic. The ID mapping functionality in UniProt was used to assign gene names to the identified proteins, and the gene set file was generated using the slimGO entries from http://systemsbiology.cau.edu.cn/agriGOv2/. The AgriGO software (Tian et al., 2017) was used for Gene Ontology (GO) enrichment analysis.

### RNA extraction, cDNA synthesis, and quantitative real-time polymerase chain reaction

RNA isolation was carried out using the TRIZOL^®^ Reagent (Fisher Molecular Biology, Trevose, U.S.A.) according to the supplier’s indications. cDNAs were obtained using the RevertAid First Strand cDNA Synthesis Kit (Thermo Fisher Scientific, Milan, Italy) according to the manufacturer’s suggestions. Quantitative real-time polymerase chain reaction (*q*RT-PCR) was performed with the Maxima SYBR Green qPCR Master Mix (2X) (Thermo Fisher Scientific) according to the supplier’s indications, using a Rotor-Gene 6000 PCR apparatus (Corbett Robotics Pty Ltd). Amplification conditions were as follows: denaturation at 95°C for 10 min, and 45 cycles of 95°C for 15 s and 60°C for 30 s and 72°C for 30 s. Oligonucleotide primers were designed using the Real-Time PCR Primer Design program Primer3Plus (https://primer3plus.com) from GenScript and further validated through the online software Oligo Analyzer (https://eu.idtdna.com/calc/analyzer), whereas target specificity was assessed through Primer-BLAST (https://www.ncbi.nlm.nih.gov/tools/primer-blast/) (**Supplementary Table S1**). Quantification was carried out using as reference genes *MtELF1a* (*ELONGATION FACTOR 1a*) (Medtr6g021805) and *MtACT* (*ACTIN*) (Medtr2g096840) for the experimental conditions (primed *versus* overprimed) used in this work. The following genes were tested: *MtDRP2B* (*DYNAMIN-RELATED PROTEIN*) (Medtr4g030140), *MtTRXm4* (*THIOREDOXIN m4*) (Medtr2g079420), *MtASPG1* (*ASPARTIC PROTEASE IN GUARD CELL 1*) (Medtr7g105850), *MtABA2* (*ABSCISIC ACID DEFICIENT 2*) (Medtr3g020670), *MtITPA* (*INOSINE TRIPHOSPHATE PYROPHOSPHORYLASE*) (Medtr1g047390), *MtRS2Z32* (*SERINE/ARGININE-RICH SPLICING FACTOR RS2Z32*) (LOC112416407), *MtAQR* (*RNA HELICASE AQUARIUS*) (Medtr1g038815.1), *MtAAG* (*ALKYL-ADENINE DNA GLYCOSILASE*) (Medtr7g093540), *MtEndoV* (*ENDONUCLEASE V*) (Medtr1g010130), and *MtPCNA* (*PROLIFERATING CELL NUCLEAR ANTIGEN*) (Medtr3g462130). The raw, background-subtracted fluorescence data provided by the Rotor-Gene 6000 Series Software 1.7 (Corbett Robotics) was used to estimate PCR efficiency (E) and threshold cycle number (C_t_) for each transcript quantification. Relative quantification of transcript accumulation was carried out as described by Pfaffl (2001). Heatmaps represent the Log_2_ fold changes (Log_2_ FC) of mean transcript expression levels or protein accumulation levels. For each transition within the experimental system, fold changes consider the ratio between the second condition of the pairwise comparison over the first condition.

### Comet assay

The alkaline version of the comet assay allows quantifying single-strand breaks (SSBs) formed from alkali‐labile sites as well as DNA–DNA or DNA–protein crosslinks (Collins, 2004). Seeds were harvested at the indicated time points, and embryo axes and embryos with radicle protrusion were isolated from the cotyledons and seed coat using a razor blade as reported by Pagano et al. (2022a). Nuclei were extracted as previously described (Ventura et al., 2014). The resulting suspension containing purified nuclei was mixed in equal volume with a solution containing 1% low-melting-point agarose (Sigma‐Aldrich) in phosphate‐buffered saline buffer (PBS: 140 mM NaCl, 2.7 mM KCl, 10 mM Na_2_HPO_4_, and 1.8 mM KH_2_PO_4_) maintained at 38°C. Two drops of the resulting suspension were then pipetted onto agarose-precoated slides and solidified on ice. Slides were incubated for 30* min* at 4°C in alkaline buffer (1 mM Na_2_EDTA, 300 mM NaOH, pH 13.0) and then electrophoresed in the same buffer for 25 min at 0.72* V* cm^−1^ in a cold chamber under dark conditions. After electrophoresis, slides were washed in 0.4 M Tris-HCl, pH 7.5, three times for 5* min*, rinsed in 70% ethanol (v/v) three times for 5 min at 4°C, and dried overnight at room temperature. Slides were stained with 20 µl of DAPI (4′,6‐diamidino‐2‐phenylindole) solution (1 mg ml^−1^, Sigma‐Aldrich). For each slide, 100 nucleoids were scored, visualized using an Olympus BX51 fluorescence microscope with an excitation filter of 340–380 nm and a barrier filter of 400 nm. Images were captured using an Olympus MagnaFire camera equipped with Olympus Cell‐F software. Nucleoids were classified as previously described by Collins (2004), where each type of nuclei morphology belongs to a class from 0 to 4. The results were expressed in arbitrary units (a.u.), calculated using the following formula: a.u. = [Σ(N_c_ × c) × 100]/N_tot_, where N_c_ is the number of nuclei of each class, c is the class number (e.g., 0, 1, 2, 3, and 4), and N_tot_ is the total number of counted nuclei (Collins, 2004).

### Statistical analyses

Statistical analysis of protein accumulation, germination test, ROS accumulation, comet assay, and *q*RT-PCR data was performed using the Student’s *t*-test. Asterisks indicate statistically significant differences determined using Student’s *t*-test (*p < 0.05; **p < 0.01, ***p < 0.001). The significance criteria for comparisons within the proteomic dataset were based on adjusted *p* < 0.05. Changes in protein or transcript levels within a given pairwise comparison were expressed as log_2_ fold change (FC), where FC equals the second condition divided by the first condition. As for proteins detected only in one condition of a given pairwise comparison, pseudo fold changes were estimated using the mean of the 10% smallest protein averages instead of the absent group average. The correlation analyses between protein accumulation and gene expression data, both expressed as log_2_ FC, were performed using the online tool developed by Mann and Whitney (1947) (http://www.statskingdom.com/170median_mann_whitney.html).

## Results

### Hydropriming resulted in synchronized seed germination

**Figure 1:**
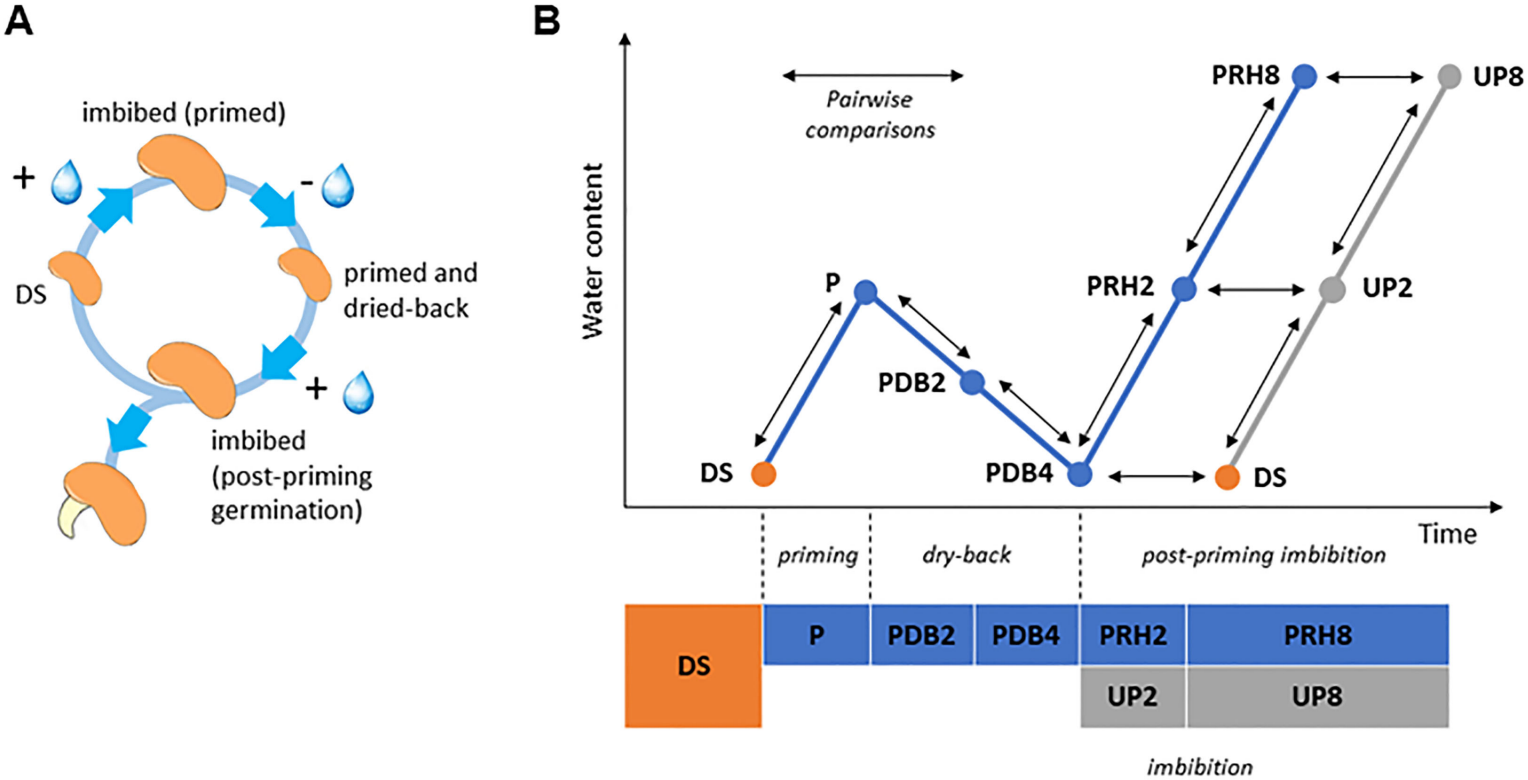
**(A)** The rehydration–dehydration cycle applied to *Medicago truncatula* seeds. **(B)** Detailed representation of the experimental system used in this study, highlighting the 10 pairwise comparisons (arrows) selected for proteomic data analysis. DS, dry seed. P, primed (hydropriming for 2 h). PDB2 and PDB4, hydropriming followed by 2 h and 4 h of dry-back, respectively. PRH2 and PRH8, primed seeds after 2 h and 8 h of rehydration, respectively (post-priming imbibition). UP, unprimed. UP2 and UP8, unprimed seeds after 2 h and 8 h of rehydration, respectively (imbibition).

UP seeds started germination within 1 day from the beginning of imbibition and reached the maximum germination percentage (82.00% ± 5.70%) at 3 days. Acceleration of the germination process was observed in P seeds, since at 1 day following post-priming imbibition germination percentage significantly increased (73.00% ± 4.47%), compared to UP seeds (47.00% ± 9.08%) (55.32% increase) (**Supplementary Figure S1**). Germination parameters are reported in **Table 1**. The positive impact of HP was evidenced by the significant decrease in *MGT* (mean germination time) (28.66% decrease) and increase in MGR (mean germination rate) (40.32% increase) and CVG (coefficient of velocity of germination) (40.90% increase) observed in P seeds compared to UP seeds (**Table 1**). No significant impact of HP on seedling biomass (fresh and dry weight, radicle length) was evidenced (**Supplementary Table S2**). The experimental system designed for the proteomic approach is representative of the rehydration–dehydration cycle (**Figure 1A**). The design includes the dry seeds (DS) and primed seeds (P) and two different time points along dry-back (2 h, PDB2 and 4 h, PDB4) (**Figure 1B**). The post-priming imbibition was monitored at 2 h (PRH2) and at 8 h (PRH8) and performed in parallel with imbibition of UP seeds monitored at 2 h (UP2) and 8 h (UP8) (**Figure 1B**). Concomitant with the progressive dehydration, ROS amounts appeared significantly higher in primed seeds at 2 h of dry-back, compared to primed imbibed seeds and seeds at the end of dry-back (**Supplementary Figure S2**). At 2 h of imbibition, the unprimed seeds showed significantly higher ROS levels compared to primed seeds (**Supplementary Figure S2**). *M. truncatula* seeds collected at different time points along the rehydration–dehydration cycle underwent proteomic analysis.

**Table 1:** Germination parameters calculated based on results of germination tests carried out on *M. truncatula* unprimed (UP) and hydroprimed (P) seeds.

Treatment	*G* (%)	*MGT* (days)	*CVG* (%)	*MGR* (day^−1^)	*U* (bit)	*Z* (unitless)
UP	82.00 ± 5.70	1.64 ± 0.15	61.51 ± 5.61	0.62 ± 0.06	1.38 ± 0.14	0.40 ± 0.08
P	83.00 ± 5.70	1.17 ± 0.17**	86.67 ± 11.65**	0.87 ± 0.12**	0.59 ± 0.35**	0.79 ± 0.17**

Asterisks indicate statistically significant differences determined using two-tailed heteroscedastic Student’s t-test (*p < 0.05; **p < 0.01; ***p < 0.001). G, Germinability. MGT, Mean germination time. CVG, Coefficient of velocity of germination. MGR, Mean germination rate. U, Uncertainty. Z, Synchronization index.

### Outline of the seed proteome along the rehydration–dehydration cycle

**Figure 2:**
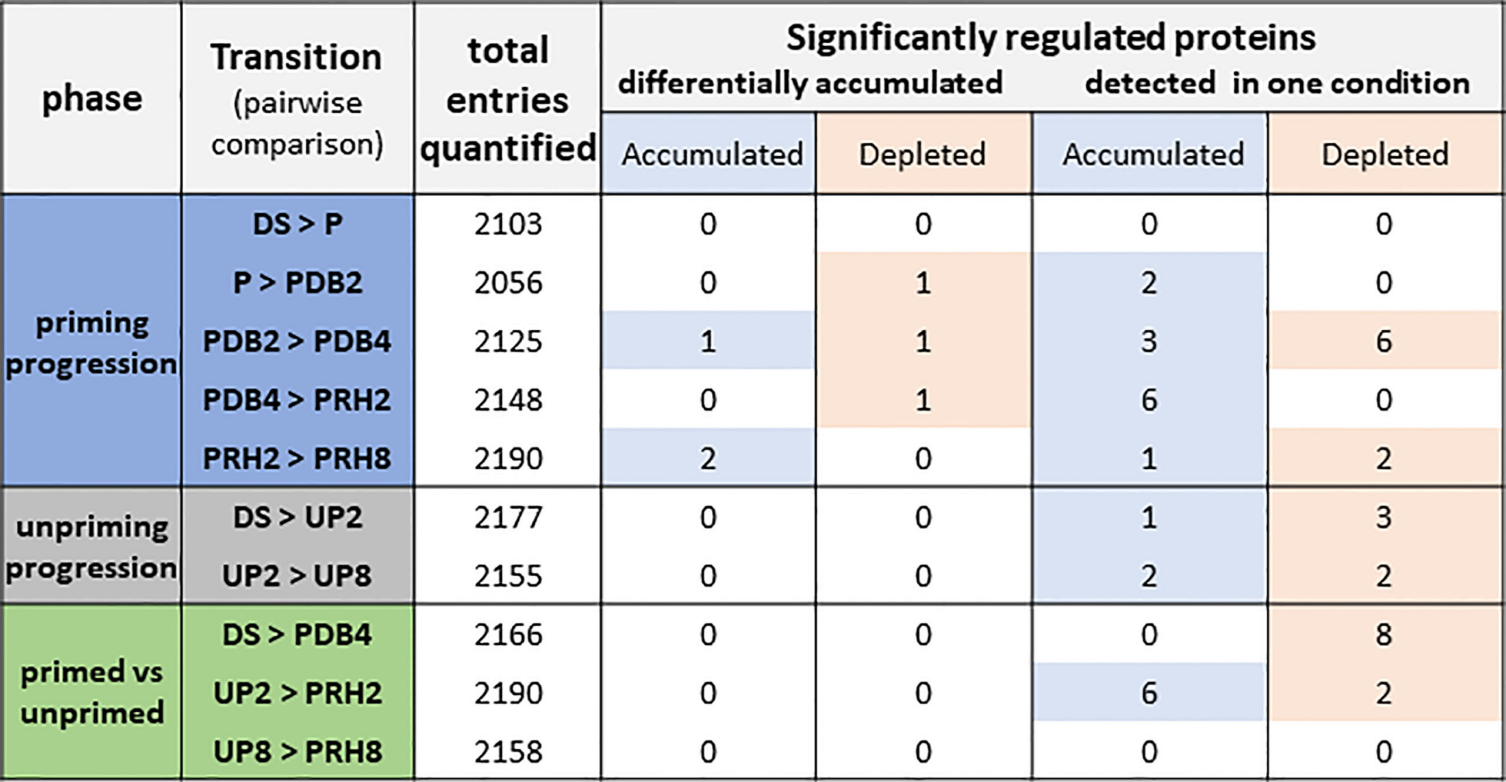
Total number of proteins identified in *Medicago truncatula* seeds along the rehydration–dehydration cycle. Three phases were considered. For each phase, the transitions or pairwise comparisons between different metabolic stages analyzed in this study are shown. The number of differentially accumulated proteins detected for each pairwise comparison and proteins detected only in one condition is also indicated. The table includes proteins that were represented by at least two peptides with a maximum of five missing values per protein. Depleted: proteins that were more represented in the first condition of the pairwise comparison. Accumulated: proteins that were more represented in the second condition of the pairwise comparison. The significance criteria included the following: adjusted *p*-value < 0.05, log2 fold change ≥ 1, and log2 fold change ≤ −1. DS, dry seed. P, primed (hydropriming for 2 h). PDB2 and PDB4, hydropriming followed by 2 h and 4 h of dry-back, respectively. PRH2 and PRH8, primed seeds after 2 h and 8 h of rehydration, respectively (post-priming imbibition). UP, unprimed. UP2 and UP8, unprimed seeds after 2 h and 8 h of rehydration, respectively (imbibition).

As shown in **Figure 2**, three different phases were considered, defined as “priming progression” (HP, dry-back, post-priming rehydration), “unpriming progression” (imbibition of unprimed seeds), and “primed *versus* unprimed”. To better understand the changes in the proteome associated with the rehydration–dehydration cycle, different transitions were considered for each phase (**Figure 2**) and regarded as pairwise comparisons between two of the different categories outlined in **Figure 1B**. Overall, the number of proteins detected in each pairwise comparison ranged from 2,056 to 2,190 (**Figure 2**, total entries quantified). Among these, the significantly regulated proteins were grouped in two distinct classes: proteins that were differentially accumulated and proteins detected only in one condition. In both cases, the significantly regulated proteins could be accumulated or depleted, depending on the pairwise comparison (**Figure 2**). The significance criteria were based on adjusted *p* < 0.05, log_2_ FC ≥ 1, and log_2_ FC ≤ −1.

The “priming progression” phase included five transitions or pairwise comparisons (DS *vs.* P, P *vs.* PDB2, PDB2 *vs.* PDB4, PDB4 *vs.* PRH2, and PRH2 *vs.* PRH8), and accounted for changes in 26 proteins, among which 11 were associated with the PDB2 *vs.* PDB4 transition (representative of the dry-back step) (**Figure 2**). The “unpriming progression” phase included two transitions or pairwise comparisons (DS *vs.* UP2, and UP2 *vs.* UP8) representative of imbibition of UP seeds and revealed changes in seven proteins (**Figure 2**). Finally, the three transitions or pairwise comparisons (DS *vs.* PDB4, UP2 *vs.* PRH2, and UP8 *vs.* PRH8), representative of the “primed *vs.* unprimed” phase, showed changes in 16 proteins, equally distributed between DS *vs.* PDB4 (pairwise comparison involving the dry seed and the primed, dried-back seed) and UP2 *vs.* PRH2 (pairwise comparison involving the UP and P seeds at 2 h of imbibition) (**Figure 2**).

### Selective enrichment of GO terms represented in the *M. truncatula* seed proteome

**Figure 3:**
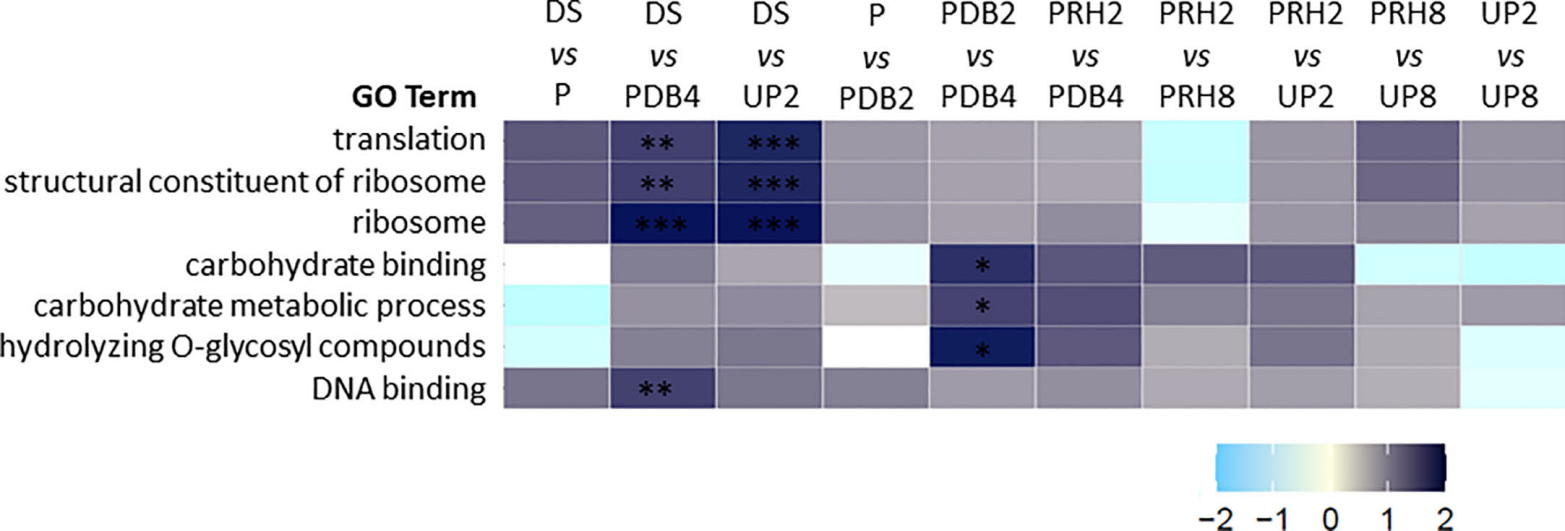
Heatmap representing normalized enrichment score relative to control (NES) obtained through gene set enrichment analysis (GSEA) including only the GO terms that were significantly (adjusted *p*-value) enriched in at least one of the pairwise comparisons. First condition *versus* (*vs*) second condition as control. The definition and the number identifier of the GO terms are provided. Adjusted *p*-value: *< 0.05, **< 0.01, ***< 0.001. NES, normalized enrichment score relative to control. Translation, GO:0006412. Structural constituent of ribosome, GO:0003735. Ribosome, GO:0005840. Carbohydrate binding, GO:0030246. Carbohydrate metabolic process, GO:0005975. Hydrolyzing O-glycosyl compounds GO:0004553. DNA binding, GO:0003677.

The AgriGO software identified and classified 577 GO terms enriched in the identified proteins. The 24 GO terms listed within the first hierarchical level are shown in **Supplementary Figure S3**. As a subsequent step, gene set enrichment analysis (GSEA) was performed to obtain the normalized enrichment score (NES) and those GO terms specifically enriched in each pairwise comparison. Results of this analysis are represented as a heatmap in **Figure 3**. Seven GO terms were significantly enriched in 3 out of the 10 comparisons considered: translation (GO:0006412), structural constituent of ribosome (GO:0003735), ribosome (GO:0005840), carbohydrate binding (GO:0030246), carbohydrate metabolic process (GO:0005975), hydrolyzing O-glycosyl compounds (GO:0004553), and DNA binding (GO:0003677) (**Figure 3**). The pairwise comparison DS > PDB4 showed four different GO categories that were differentially enriched: translation (GO:0006412), structural constituent of ribosome (GO:0003735), ribosome (GO:0005840), and DNA binding (GO:0003677) (**Figure 3**). In the case of pairwise comparison DS > UP2, three significantly enriched GO categories were highlighted, namely, translation (GO:0006412), structural constituent of ribosome (GO:0003735), and ribosome (GO:0005840). Finally, the pairwise comparison PDB2 > PDB4 showed three categories significantly enriched: carbohydrate binding (GO:0030246), carbohydrate metabolic process (GO:0005975), and hydrolyzing O-glycosyl compounds (GO:0004553). No significantly enriched GO categories were identified in the other considered pairwise comparisons. Results of this analysis allowed the selection of the GO terms showing significant changes along the rehydration–dehydration cycle, a useful information for a deeper understanding of the function of the differentially regulated proteins disclosed by proteomics.

### Proteome changes along dry-back involve proteins with roles in desiccation tolerance

**Figure 4:**
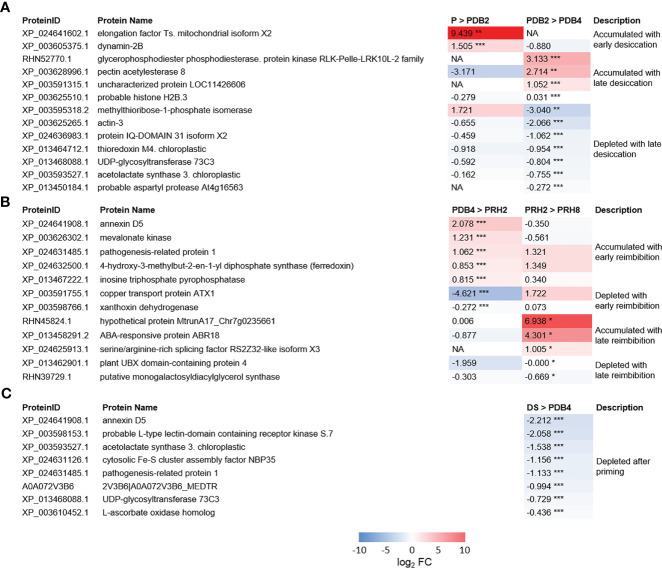
**(A)** Heatmaps displaying changes in protein accumulation at two different transitions along dehydration in *M. truncatula* seeds. **(B)** Heatmaps displaying changes in protein accumulation at two different transitions along rehydration. **(C)** Heatmaps displaying changes in protein accumulation between unprimed dry seed and dehydrated seeds after priming. Values are expressed as log_2_ fold change of the second condition divided by the first condition of the pairwise comparison. DS, dry seed; P, primed seeds; PDB2, primed seeds after 2 h dry-back; PDB4, primed seeds after 4 h dry-back; PRH2, primed seeds after 4 h dry-back and 2 h reimbibition; PRH8, primed seeds after 4 h dry-back and 8 h reimbibition, NA, not accumulated. Asterisks indicate statistically significant differences as determined through two-tailed Student’s *t*-test. As for gene expression analysis *p < 0.05; **p < 0.01; ***p < 0.001.

As shown in **Figure 4**, the protein Dynamin 2B (XP_003605375.1) was accumulated *ex novo* in *M. truncatula* primed seeds exposed to 2 h of desiccation (detected only in PDB2). Actin 3 (XP_003625265.1) was found at 2 h of dry-back (PDB2) and subsequently depleted at 4 h of dry-back (PDB4) (**Figure 4**). Thioredoxin m4 (XP_013464712.1), IQ DOMAIN 31 isoform X2 protein (XP_024636983.1), a cyclic nucleotide-gated channel (CNGC) regulated by calmodulin, and the histone variant H2B.3 (XP_003625510.1) were also depleted in PDB4 seeds (**Figure 4**). At the end of dry-back (PDB4), other proteins appeared *ex novo* (**Figure 4**), namely, RLK-Pelle-LRK10L-2 protein kinase (RHN52770.1) and aspartic protease (XP_013450184.1) (**Figure 4**).

### The proteome of primed dried-back versus dry seeds

Five proteins identified in the dry seeds (DS) were depleted in the PDB4 P seeds (**Figure 4**). One of these proteins was annexin D5 (XP_024641908.1), a calcium ion binding protein involved in various cellular processes such as actin binding, maintenance of vesicular trafficking, cellular redox homeostasis, and ion transport. Also, the pathogenesis-related protein 1 (XP_024631485.1) and the L-type lectin-domain containing receptor kinase S.7 (LECRK-S.7) (XP_003598153.1) were depleted in PDB4 seeds (**Figure 4**). The list of depleted proteins also includes the cytosolic Fe-S cluster assembly factor NBP35 (Nucleotide binding protein 35) (XP_024631126.1) and L-ascorbate oxidase (XP_003610452.1) (**Figure 4**).

### Changes in *M. truncatula* seed proteome during post-priming imbibition

The differentially regulated proteins identified during post-priming imbibition are shown in **Figure 4**. Five proteins were accumulated *ex novo* at 2 h of post-priming imbibition (PRH2), namely, annexin D5 (XP_024641908.1), pathogenesis-related protein 1 (PR1) (XP_024631485.1), mevalonate kinase (XP_003626302.1), xanthoxin dehydrogenase (XP_003598766.1), and inosine triphosphate pyrophosphorylase (ITPA) (XP_013467222.1). ITPA cleaves inosine triphosphate (ITP) and xanthine triphosphate (XTP) as well as their deoxyribose forms into monophosphates. The serine/arginine-rich (SR) splicing factor RS2Z32-like isoform X3 (SRSF RS2Z32-like isoform X3) (XP_024625913.1) accumulated in PRH8 seeds (**Figure 4**).

### Changes in transcript levels and correlation with protein abundance

**Figure 5:**
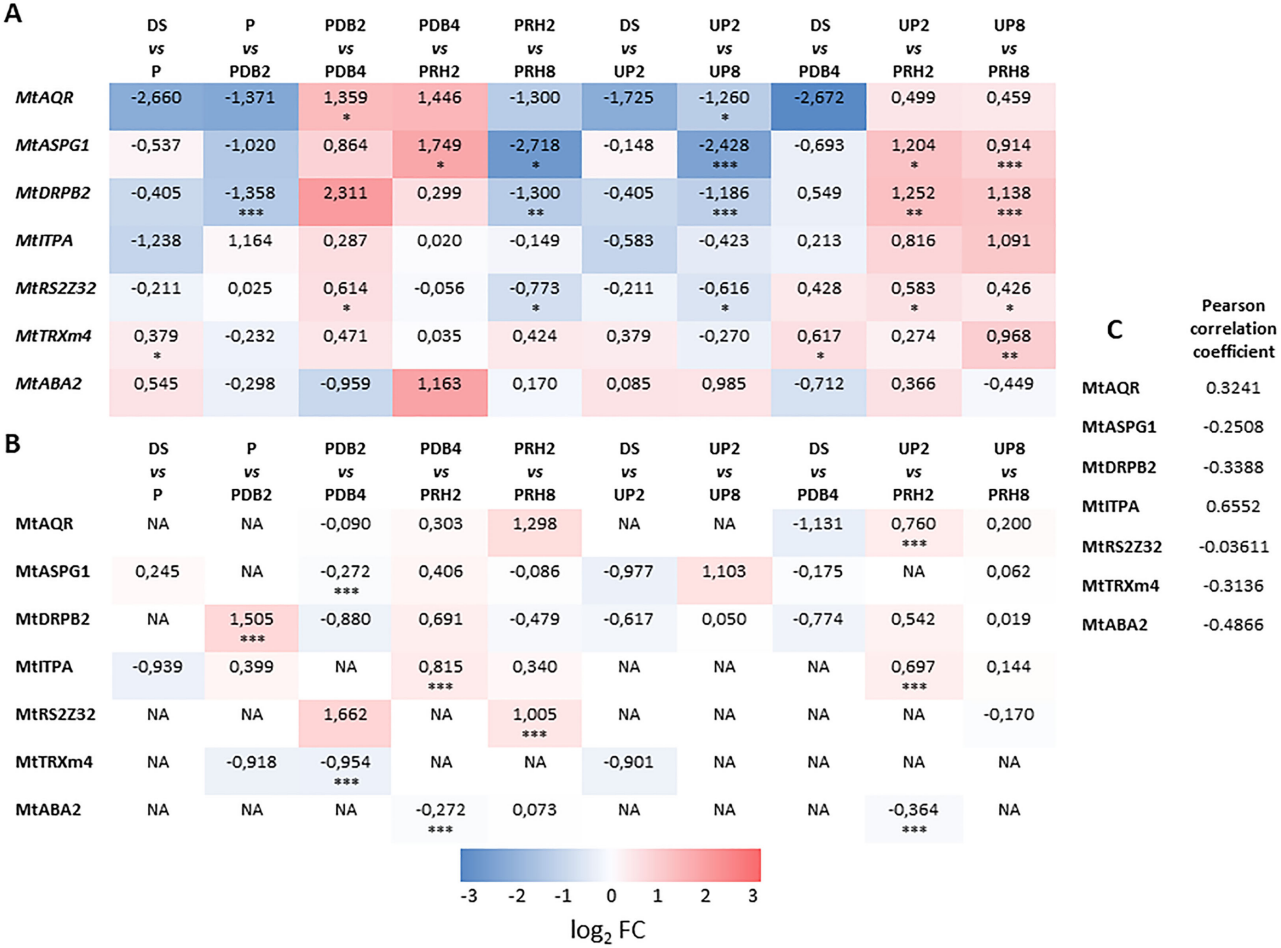
**(A)** Heatmap displaying changes in gene expression at different transitions along and between priming and control treatments of *M. truncatula* seeds. **(B)** Heatmap displaying changes in protein levels at different transitions along and between priming and control treatments of *M. truncatula* seeds. Values are expressed as log_2_ fold change of the second condition divided by the first condition of the pairwise comparison. DS, dry seed; P, primed seeds; PDB2, primed seeds after 2 h dry-back; PDB4, primed seeds after 4 h dry-back; PRH2, primed seeds after 4 h dry-back and 2 h reimbibition; PRH8, primed seeds after 4 h dry-back and 8 h reimbibition; UP2, unprimed seeds after 2 h imbibition; UP8, unprimed seeds after 8 h imbibition. Asterisks indicate statistically significant differences as determined through two-tailed Student’s *t*-test. As for gene expression analysis, *p < 0.05; **p < 0.01; ***p < 0.001. **(C)** Comparative analysis showing the Pearson correlation coefficient between the log_2_ FC transcript and protein level of the candidate genes. *Mt*, *Medicago truncatula*. *AQR, RNA HELICASE AQUARIUS. ASPG*, *ASPARTIC PROTEASE IN GUARD CELL*. *DRP*, *DYNAMIN-RELATED PROTEIN*. *ITPA*, *INOSINE TRIPHOSPHATE PYROPHOSPHORYLASE*. *RS2Z32, SERINE/ARGININE-RICH SPLICING FACTOR. TRX*, *THIOREDOXIN*. *ABA*, *ABSCISIC ACID DEFICIENT*. NA, not accumulated.

Seven proteins differentially accumulated along the rehydration–dehydration cycle were selected for further characterization. MtDRP2B, MtTRXm4, and MtASPG1 proteins, showing changes in *M. truncatula* seeds challenged with dehydration stress, were selected due to their possible involvement in desiccation tolerance. MtITPA, MtABA2, MtRS2Z32, and MtAQR proteins were chosen since they were differentially regulated during post-priming imbibition. Changes in transcript levels were assessed using *q*RT-PCR analysis, and the relationship between protein abundance and the corresponding gene transcript level was evaluated. The results of the *q*RT-PCR experiments and the protein accumulation patterns are illustrated as heatmaps (**Figure 5**).

The heatmap representing changes in transcript levels (**Figure 5A**) revealed a variegated pattern of statistically significant upregulations and downregulations associated with the different transitions or pairwise comparisons. The *MtAQR* gene showed upregulation in the PDB2 *vs.* PDB4 transitions whereas downregulation occurred in the UP2 *vs.* UP8 transitions. The *MtASPG1* transcript was accumulated in the PDB4 *vs.* PRH2, UP2 *vs.* PRH2, and UP8 *vs.* PRH8 transitions. A decrease in *MtASPG1* transcript levels was associated to PRH2 *vs.* PRH8 and UP2 *vs.* UP8 transitions (**Figure 5A**). As for the *MtDRPB2* gene, upregulation was observed in UP2 *vs.* PRH2 and UP8 *vs.* PRH8, whereas downregulation occurred in P *vs.* PDB2, PRH2 *vs.* PRH8, and UP2 *vs.* UP8. With respect to the *MtRS2Z32* gene, upregulation was detected in the PDB2 *vs.* PDB4, UP2 *vs.* PRH2, and UP8 *vs.* PRH8 transitions (**Figure 5A**). Downregulation of *MtRS2Z32* gene occurred in the PRH2 *vs.* PRH8 and UP2 *vs.* UP8 transitions. Interestingly, the *MtTRXm4* gene showed upregulation only in the DS *vs.* P, DS *vs.* PDB4, and UP8 *vs.* PRH8 transitions. Finally, no significant changes in transcript levels were observed throughout the rehydration–dehydration cycle for *MtITPA* and *MtABA2* genes (**Figure 5A**).

Overall, results of *q*RT-PCR analysis highlighted the potential role of the tested genes in the seed response to priming. However, to what extent could such transcriptional profiles correlate with the accumulation patterns of their encoded proteins? In order to address this question, a heatmap representative of changes in protein levels has been assembled (**Figure 5B**). The MtAQR protein was accumulated in the UP2 *vs.* PRH2 transition. The MtASPG1 protein level was lowered in the PDB2 *vs.* PDB4 transition. As for the MtDRPB2 protein, accumulation was observed in the P *vs.* PDB2 transition. With respect to the MtITPA protein, accumulation was detected in PDB4 *vs.* PRH2 and UP2 *vs.* PRH2, whereas MtRS2Z32 was accumulated in association with PRH2 *vs.* PRH8. The level of MtTRXm4 and MtABA2 proteins was decreased in PDB2 *vs.* PDB4 and PDB4 *vs.* PRH2. Based on the comparative analysis (Pearson’s correlation coefficient) between the expression patterns of the candidate genes and protein accumulation level, no significant correlation was found (**Figure 5C**). Such findings highlight the complexity of the regulatory levels that rule the different players disclosed in this proteomic study.

### Does ITPA contribute to the response of primed seeds to genotoxic stress? A proof of concept

**Figure 6:**
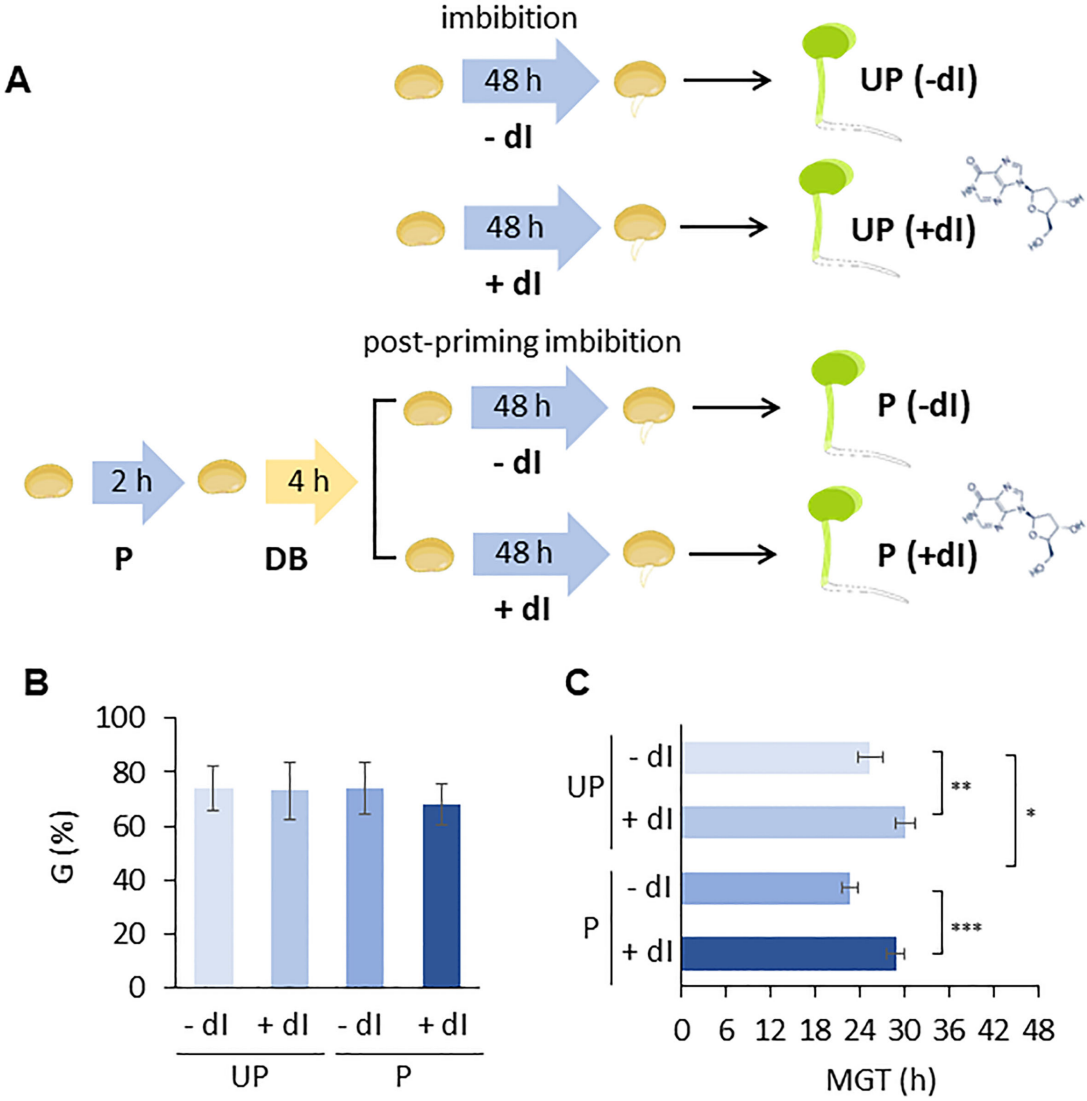
**(A)** Experimental system designed to test the improved ability of primed *M. truncatula* seeds to withstand genotoxic stress induced in the presence of 20 mM deoxy-inosine (dI), compared to unprimed seeds. **(B)** Germinability (*G*) calculated for unprimed and primed *M. truncatula* seeds in the presence/absence of dI. **(C)** Mean germination time (*MGT*). Asterisks indicate statistically significant differences as determined through two-tailed heteroscedastic Student’s *t*-test comparing UP − dI *vs.* P − dI, UP + dI *vs.* P + dI, UP − dI *vs.* UP + dI, and P − dI *vs.* P + dI, *p < 0.05, **p < 0.01, ***p < 0.001. UP, unprimed. P, primed. DB, dry-back. − dI, absence of deoxy-inosine. + dI, 20 mM deoxy-inosine.

**Figure 7:**
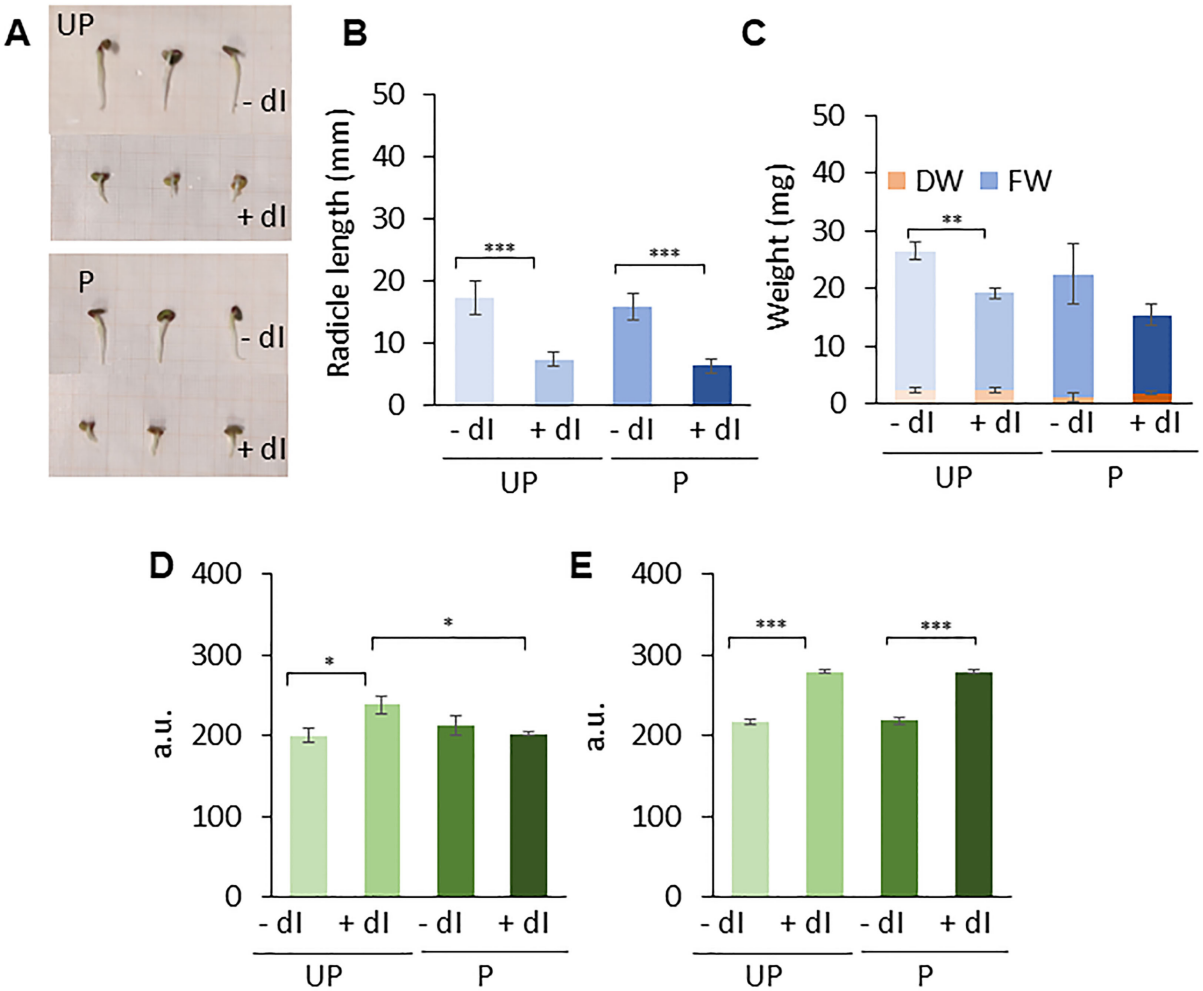
Phenotyping and comet assay analyses. **(A)** *M. truncatula* untreated and primed seeds at 48 h following the beginning of imbibition in the presence/absence of 20 mM dI. **(B)** Radicle length. **(C)** Biomass measured as fresh weight (FW) and dry weight (DW), respectively. Alkaline comet assay was used to measure DNA strand break accumulation in *M. truncatula* untreated and primed seeds at 24 h **(D)** and 48 h **(E)** of imbibition in the presence/absence of 20 mM dI. Values are expressed as mean ± SD of three independent replications with 20 seeds for each replication. Asterisks indicate statistically significant differences as determined through two-tailed heteroscedastic Student’s *t*-test comparing UP − dI *vs.* P − dI, UP + dI *vs.* P + dI, UP − dI *vs.* UP + dI, and P − dI *vs.* P + dI, *p < 0.05, **p < 0.01, ***p < 0.001. UP, unprimed. P, primed. DB, dry-back. − dI, absence of deoxy-inosine. + dI, 20 mM deoxy-inosine.

An experimental system was designed in which P and UP *M. truncatula* seeds were imbibed in the presence/absence of exogenous 2’-deoxyinosine (dI) and the resulting germination performance was evaluated (**Figure 6A**). In order to select the proper dI concentration, a preliminary analysis was carried out by testing different dI doses in the 0–40 mM range. Increasing dI concentrations did not affect germinability (*G*) (**Supplementary Figure S4A**) whereas germination speed progressively decreased, as evidenced by the estimated T_50_ values (**Supplementary Figure S4B**). A significant dose-dependent decrease in radicle length was observed in 4-day-old seedlings developed from seeds exposed to dI (**Supplementary Figure S4C**). The dose-dependent growth impairment observed in seedlings treated with dI is shown in **Supplementary Figure S5**. The 20 mM dI dose, resulting in a distinctive phenotype in terms of germination performance and seedling phenotype, was selected for further analyses. The effects of HP on the seed ability to germinate in the presence/absence of 20 mM dI are reported in **Figures 6B, C**. No significant differences were observed in germinability of UP seeds when treated with or without 20 mM dI. A similar response was observed for the P seeds (**Figure 6B**). Exposure to dI resulted in reduced germination speed, as evidenced by the estimated MGT values, in both UP and P seeds (**Figure 6C**), impairing the beneficial effects of HP. A significant decrease in radicle length was observed at 48 h from the beginning of imbibition in both UP and P seeds exposed to 20 mM dI (**Figures 7A, B**). The effects of the treatment were visible when looking at the 4-day-old seedling phenotype (**Supplementary Figure S6**). Exposure to dI caused a significant decrease in the fresh biomass of seedlings developed from UP seeds (**Figure 7C**). Such decrease was maintained also in seedlings derived from P seeds exposed to dI (**Supplementary Figure S6**).

The genotoxic damage induced by dI was assessed using the alkaline comet assay (**Figures 7D, E**). DNA strand break accumulation was measured in *M. truncatula* UP and P seeds collected at 24 h and 48 h of imbibition in the presence/absence of 20 mM dI. At 24 h of imbibition, a significant accumulation of DNA damage is observed only in the UP seeds exposed to dI compared to untreated UP seeds. No significant differences in DNA strand break levels were detected in P seeds, independent of treatments (**Figure 7D**). Subsequently, at 48 h, both UP and P seeds showed comparable DNA damage accumulation, in the presence/absence of dI (**Figure 7E**). Such a pattern suggests that at 24 h, P seeds can better cope in terms of dI-induced DNA damage accumulation, compared to UP seeds, whereas at 48 h, such beneficial effects are no longer evident.

**Figure 8:**
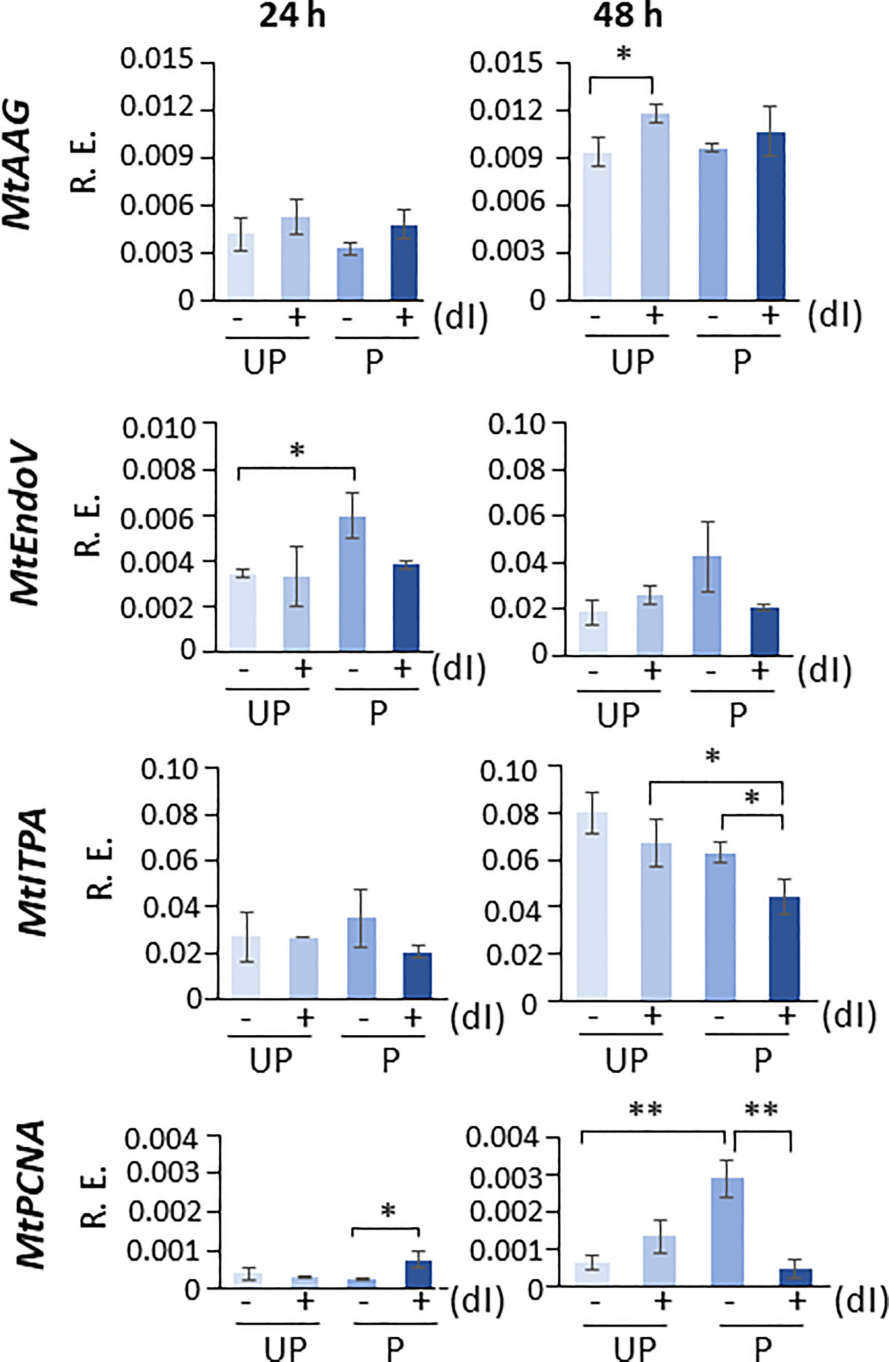
Expression profiles of *MtAAG*, *MtEndoV*, *MtITPA*, and *MtPCNA* genes measured by *q*RT-PCR in *M. truncatula* untreated and primed seeds at 24 h and 48 h of imbibition in the presence/absence of 20 mM dI. Asterisks indicate statistically significant differences as determined through two-tailed heteroscedastic Student’s *t*-test comparing UP − dI *vs.* P − dI, UP + dI *vs.* P + dI, UP − dI *vs.* UP + dI, and P − dI *vs.* P + dI, *p < 0.05, **p < 0.01, ***p < 0.001. UP, unprimed. P, primed. DB, dry-back. − dI, absence of deoxy-inosine. + dI, 20 mM deoxy-inosine. R.E., relative expression. AAG, alkyl-adenine glycosylase. ENDO, endonuclease. ITPA, inosine triphosphate pyrophosphorylase. PCNA, proliferating cell nuclear antigen.

**Figure 9:**
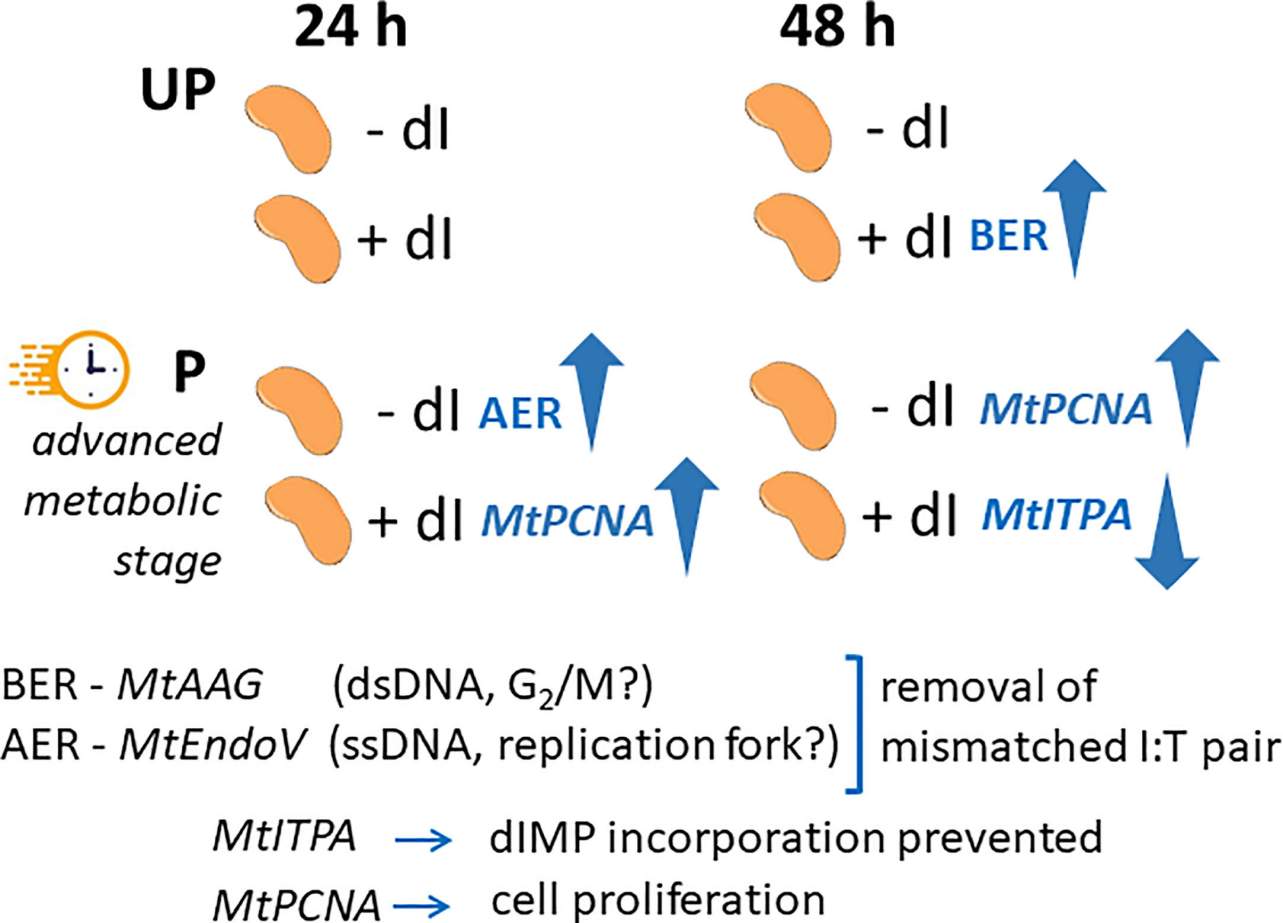
Schematic representation integrating the temporal dynamics of the seed response to dI in terms of gene expression profiles. The comparison between UP and P seeds is highlighted in view of the different physiological stage. UP, unprimed. P, primed. DB, dry-back. − dI, absence of deoxy-inosine. + dI, 20 mM deoxy-inosine. AAG, alkyl-adenine glycosylase. ENDO, endonuclease. ITPA, inosine triphosphate pyrophosphorylase. PCNA, proliferating cell nuclear antigen. dsDNA, double-stranded DNA. ssDNA, single-stranded DNA. BER, base excision repair. AER, alternative excision repair.

In order to highlight the potential role of specific molecular players that could contribute to the ability of P seeds to repair the dI-induced DNA damage, the expression profiles of *MtAAG* and *MtEndoV* genes, involved in the repair of the mismatched I:T pair, were evaluated. *MtAGG* encodes the BER enzyme alkyl-adenine DNA glycosylase that removes the hypoxanthine base from DNA as well as other purines modified by alkylation and oxidative damage. *MtEndoV* encodes the repair enzyme deoxyinosine 3’ endonuclease that recognizes deoxyinosine in double- and single-stranded DNA and cleaves the second phosphodiester bond 3’ to the mismatched I:T pair, leaving a nick with 3’-hydroxyl and 5’-phosphate groups. EndoV is part of the alternative excision repair (AER) pathway that only produces a single nick on one side of the damaged base and does not directly remove deoxyinosine from the DNA strand. As shown in **Figure 8**, the expression profiles of *MtAAG* and *MtEndoV* genes were tested at 24 h and 48 h of imbibition. At the earliest time point, the only response observed was a significant increase in the *MtEndoV* transcript levels, occurring in the untreated P seeds. A significant accumulation of *MtAAG* mRNA was detected only in UP seeds treated with dI at 48 h of imbibition (**Figure 8**). Such findings highlight a significant, although slight, repair response of the *M. truncatula* seeds mediated by these BER and AER genes. The latter might be responsive to the different conditions of the experimental system, considering that priming brings seeds towards a more advanced physiological stage, closer to the end of germination. The expression of *MtITPA* gene was significantly decreased in P seeds exposed to dI at 48 h, and this was the only change observed (**Figure 8**). In order to gain information on the cell proliferation activity at the two tested time points, the *MtPCNA* gene was used as cell division marker. At 24 h, a significant enhancement of *MtPCNA* mRNA levels occurred in P seeds challenged with dI, while at 48 h, the highest expression level was observed in the untreated P seeds (**Figure 8**). In the attempt to integrate the information gathered at different levels, using comet assay and *q*RT-PCR, a working hypothesis is presented in **Figure 9** and critically analyzed in the Discussion section.

## Discussion

The present study provides a proteome-wide fingerprint of the rehydration–dehydration cycle in *M. truncatula* seeds. It is worth noting that the term “rehydration–dehydration cycle” has been adopted in this study in order to better underline the progression of the treatment (imbibition of the dry seed, desiccation, and finally re-imbibition leading to germination) in terms of desiccation stress and repair activity associated with the pre-germinative metabolism. Alternative hydration and rehydration treatments applied once or twice to seeds, using water without aeration, are also referred to as “hardening” (Farooq et al., 2019). The changes hereby observed in the proteome of *M. truncatula* seeds along dry-back involve proteins with roles in desiccation tolerance, suggesting some sensitive cellular and subcellular targets. Among these, the dynamin-related protein 2B, accumulated *ex novo* during dry-back, was previously associated with the response to desiccation-induced damage (Bednarek and Backues, 2010; Boissan et al., 2014; Couchoud et al., 2019). Other distinctive features of the dry-back proteome include depletion of actin-3 possibly reflecting cytoskeleton disassembly induced by dehydration (Sniegowska-Swierk et al., 2015). The protein changes occurring in dried-back seeds suggest for the contribution of different metabolic pathways, e.g., redox regulation (thioredoxin m4; Courteille et al., 2013; Kang and Wang, 2016), IQ DOMAIN 31 isoform X2 protein (a cyclic nucleotide-gated channel regulated by calmodulin) (Fischer et al., 2013), and the histone variant H2B.3 (Jiang et al., 2020). At the same time, different proteins appeared *ex novo* in dried-back seeds, such as RLK-Pelle-LRK10L-2 protein kinase that is part of a protein family involved in cell wall-associated processes (Lehti-Shiu et al., 2009; Ma et al., 2015), and aspartic protease with roles in drought stress response (Kollipara et al., 2002), osmopriming (Kubala et al., 2015), and the regulation of dormancy, viability, and germination of Arabidopsis seeds (Shen et al., 2018).

The comparison of PDB4 *versus* DS has highlighted the depletion of five proteins already found in DS, among which is annexin D5, a calcium-binding protein involved in actin binding, maintenance of vesicular trafficking, membrane stability, cellular redox homeostasis, and ion transport (Lin et al., 2020). It is difficult to explain the role of the PR1 protein along the rehydration–dehydration cycle of *M. truncatula* seeds. However, downregulation of *PR1* gene has been reported in *Brassica napus* L. subjected to salt stress (Zbiri et al., 2021). NBP35 helps in the assembly of Fe-S clusters by acting as a scaffold and forming a homodimer. Arabidopsis embryo development was aborted at early stage in the absence of NBP35, thus suggesting a role in germination (Bernard et al., 2013; Bych et al., 2008). The L-type lectin-domain containing receptor kinase S.7 belongs to a class of plasma membrane-localized receptor-like kinases (RLKs) with roles in perceiving environmental or development signals and regulating plant development (Sun et al., 2020). The depletion of the above-mentioned proteins might reflect the advanced physiological stage of the primed and dried-back seeds, compared to the unprimed dry seeds.

Another key point of this study deals with the seed response during post-priming imbibition. Based on the reported data, primed seeds were equipped by specific proteins that appeared *ex novo* at 2 h and 8 h of imbibition. Accumulation of xanthoxin dehydrogenase, an oxidoreductase involved in ABA biosynthesis and regulation, could represent potential hallmark of the enhanced stress tolerance provided by priming. The conversion of xanthoxin to abscisic aldehyde catalyzed by this oxidoreductase is one of the last steps of ABA biosynthesis, crucial for the basal level of the phytohormone (Leon-Kloosterziel et al., 1996). Transcriptional activation of the *ABA2* gene, encoding xanthoxin dehydrogenase, correlated with acquisition of desiccation tolerance by the intermediate coffee seeds (Dussert et al., 2018), whereas knock-out of the rice *OsABA2* gene disrupted the ABA/GA ratio with impact on seed germination and dormancy as well as enhanced H_2_O_2_ accumulation (Liao et al., 2018). The observed accumulation of xanthoxin dehydrogenase in P *M. truncatula* seeds at the onset of germination might contribute to enhance the resilience level of embryo and seed tissues exposed to unexpected drought conditions. Accumulation of mevalonate kinase in the lettuce seeds proved to be a positive regulator for seed germination (Wang et al., 2015b). ITPA cleaves inosine triphosphate (ITP) and xanthine triphosphate (XTP) as well as their deoxyribose forms into monophosphates, removing the non-canonical ITP and XTP precursors, avoiding mutations deleterious to genome stability (Menezes et al., 2012) whereas the serine/arginine-rich (SR) splicing factor RS2Z32-like isoform X3 participates in abiotic stress tolerance (Duque, 2011).

Is there a gain if we compare the proteome of P *versus* UP seeds? ITPA and RNA helicase aquarius isoform X1 were detected only in the primed seeds, at 2 h of post-priming imbibition (PRH2). ITPA cleaves ITP and XTP as well as their deoxyribose forms into monophosphates, thus removing the non-canonical ITP and XTP precursors, avoiding mutations that are deleterious to genome stability (Menezes et al., 2012). ITPA has been investigated in animal cells for its relevant contribution to genome maintenance (Abolhassani et al., 2010; Yoneshima et al., 2016). Deoxyinosine (dI) is a non-canonical nucleoside containing hypoxanthine that derives from the oxidative deamination of adenine or through the action of adenosine deaminase. Deoxyinosine triphosphate (dITP) can be misincorporated into DNA leading to genome instability. ITPA hydrolyzes dITP and other inosine nucleotides preventing genotoxic damage (Sakumi et al., 2010). Lack of ITPA function in animal cells leads to dITP accumulation and incorporation into nuclear DNA during replication, finally resulting in enhanced levels of SSBs (Abolhassani et al., 2010). These findings were further corroborated by Yoneshima et al. (2016) who investigated the effects of exogenous dI added to the culture medium of ITPA-defective human cell lines. Straube et al. (2022) recently investigated the Arabidopsis *ipta* mutants, showing that the enzyme contributes to safeguarding nucleic acids from the incorporation of aberrant purine nucleotides. The evolutionarily conserved RNA helicase MAC7 is the homolog of the human MAC7 or Aquarius helicase. It is a component of the MOS4-ASSOCIATED COMPLEX (MAC), required to promote microRNA biogenesis and pre-mRNA splicing, with roles in plant development and stress response (Jia et al., 2017). It is worth noting that, in human cells, the RNA helicase aquarius participates in the DNA damage response (Sakasai et al., 2017). Interestingly, Niñoles et al. (2022) highlighted the possible contribution of the MAC complex in Arabidopsis seed quality. Enhanced expression of *MAC3A* and *MAC3B* genes in long-lived seeds suggested that changes in alternative splicing of MAC-targeted genes might result in improved germination performance (Niñoles et al., 2022). These two proteins required for genome stability and differentially accumulated in the primed *M. truncatula* seeds represent novel intriguing targets to explore the dynamics of the genotoxic stress response in the pre-germinative metabolism. *Ex novo* occurrence of ITPA protein at 2 h of post-priming imbibition did not correlate with the *MtITPA* mRNA accumulation profiles. On the other hand, the *AtITPA* transcript was found to accumulate only at 12 h of imbibition in the radicle of germinating Arabidopsis seeds (**Supplemental Figure S7**). *MtITPA* gene upregulation might take place later during germination, a limiting feature for a potential hallmark of seed quality. Differently, upregulation of *MtAQR* occurring during dry-back makes this gene useful for monitoring the seed response to desiccation, possibly in association with other genes.

Does post-priming accumulation of ITPA protein in *M. truncatula* imbibed seeds contribute to genome stability required to improve seed quality? A proof of concept was set to address this question using dI as genotoxic agent and DNA damage was assessed by alkaline comet assay. Interestingly, significant DNA damage accumulation was detected at 24 h of imbibition only in the UP *M. truncatula* seeds exposed to dI. The primed seeds represent an advanced physiological stage, compared to the untreated ones. Thus, it is possible that they differ in terms of DNA repair response already at 24 h of post-priming imbibition in the presence of dI. This is consistent with the observed decrease in T_50_ and MGT values. The effects of the genotoxic agent on DNA integrity were evident at 48 h of imbibition in both UP and P seeds, when impairment in radicle development was also observed. This might be explained considering the prolonged exposure to dI and its action on the actively dividing radicle cells. How the UP and P *M. truncatula* seeds cope with the dI-induced DNA damage, and to what extent their different physiological state would influence their germination performance? Attention was focused on those pathways, BER and AER, involved in the removal of the mismatched I:T pair, looking at the expression dynamics of their representative *MtAGG* and *MtEndoV* genes. Such dynamics were integrated with the expression profiles of the *MtITPA* gene, required to prevent aberrant dIMP incorporation, and the *MtPCNA* gene selected as a cell proliferation marker. The schematic representation shown in **Figure 9** suggests that the BER- and AER-mediated response might apparently be influenced by the seed physiological stage. It has been hypothesized that BER and AER pathways might be differently activated, based on endogenous or exogenous signals: the AAG enzyme might be used to remove dI from double-stranded DNA in the G_2_/M phase whereas EndoV might target dI on the single-stranded DNA at the replication fork (Yao et al., 1994; Lee et al., 2009; Kuraoka, 2015). At the moment, it is difficult to validate such hypothesis in the working system hereby investigated. Another level of analysis deals with the prevention of aberrant dIMP incorporation controlled by the ITPA protein, significantly accumulated during post priming imbibition in *M. truncatula* seeds. The poor dynamics of *MtITPA* gene expression in response to dI seem to strengthen the idea that such gene does not represent a suitable hallmark of seed quality. The advanced physiological stage of HP seeds implies an increased proliferation activity, confirmed by the *MtPCNA* marker gene. Such a working system deserves a deeper investigation by expanding the analysis to a wider range of genes involved in the genotoxic stress response and possibly exploring other models of pre-germinative metabolism within legumes and in valuable horticultural and cereal model plants.

## Conclusions

In this work, the *M. truncatula* seed proteome has disclosed a peculiar aspect of the seed repair response, providing evidence of the role of a specific player (ITPA) and mechanisms, such as prevention of aberrant nucleotide incorporation, in genome maintenance. Would it be possible to use such information as a starting point to build a specific network of events entailing not only a better understanding of the seed repair response but also the identification of novel potential seed quality hallmarks? How far could such knowledge be translated to other model and crop plants? The experimental system developed in this study to investigate the response of primed seeds to the genotoxic agent deoxy-inosine might be used to screen other compounds known to trigger the activity of some of the other proteins that were differentially accumulated in the *M. truncatula* seeds along the rehydration–dehydration cycle. Definitely, concerted, multidisciplinary efforts will be necessary to address these research questions. The transition of seed priming towards improved technological levels relies on tools and knowledge allowing one to tackle the main sources of variability that impair the efficacy of treatments.

## Data availability statement

The datasets presented in this study can be found in online repositories. The names of the repository/repositories and accession number(s) can be found below: http://www.proteomexchange.org/, PXD031336.

## Author contributions

AP, AM, AB, and SSA conceived and designed the study. AP, LK, AD, SSG, FS, and HW performed the experiments. AB and AP wrote the manuscript. AP, AM, AB, SA, LK, AD, SSG, FS, and HW reviewed the manuscript. All authors contributed to the article and approved the submitted version.
