# Supplementary material for: Changes in Medicago truncatula seed proteome along the rehydration–dehydration cycle highlight new players in the genotoxic stress response
Source: Front Plant Sci. 2023 Jun 13;14:1188546. doi: 10.3389/fpls.2023.1188546 (PMC10319343; doi:10.3389/fpls.2023.1188546)
Supplement: Supplementary file 2 [file DataSheet_1.pdf]

Supplemental Data

Changes in *Medicago truncatula* seed proteome along the rehydration-dehydration cycle highlight new players in the genotoxic stress response

Andrea Pagano, Laura Kunz, Antje Dittmann, Susana De Sousa Araújo, Anca Macovei, Shraddha Shridhar Gaonkar, Federico Sincinelli, Hisham Wazeer, Alma Balestrazzi

Table S1. List of oligonucleotide primers used for *q*RT-PCR analyses. For each oligonucleotide set, PCR efficiency (E) is reported. *Mt*, *Medicago truncatula*. *ELF*, *ELONGATION FACTOR*. *ACT*, *ACTIN*. *DRP*, *DYNAMIN-RELATED PROTEIN*. *TRX*, *THIOREDOXIN*. *ASPG*, *ASPARTIC PROTEASE IN GUARD CELL*. *ABA*, *ABSCISIC ACID DEFICIENT*. *ITPA*, *INOSINE TRIPHOSPHATE PYROPHOSPHORYLASE*. *RS2Z32*, *SERINE/ARGININE-RICH SPLICING FACTOR*. *AQR*, *RNA HELICASE AQUARIUS*. *AAG*, *ALKYL-ADENINE DNA GLYCOSILASE*. *ENDO*, *ENDONUCLEASE*. *PCNA*, *PROLIFERATING CELL NUCLEAR ANTIGEN*.

Gene (Accession number)	Oligonucleotide Forward	Oligonucleotide Reverse	E
***MtELF1a* (*Medtr6g021805*)**	5’-GACAAGCGTGTGATCGAG-3’	5’-TTTCACGCTCAGCCTTAA-3’	1.73
***MtACT* (*Medtr2g096840*)**	5’-TCAATGTGCCTGCCATGTATG-3’	5’-ACTCACACCGTCACCAGAATC-3’	1.74
***MtASPG1* (*Medtr7g105850*)**	5’-GCTGGATTTGGTCGTGGAG-3’	5’-GCAGTGTTGTTCGTTGATGG-3’	1.81
***MtDRPB2* (*Medtr4g030140*)**	5’-GGAGAGCAGAGAGTGAAAGC-3’	5’-CCCAGAAAGAAGGTTAGGCA-3’	1.8
***MtITPA* (*Medtr1g047390*)**	5’-TCTGGAAAAACACTGGGAAAGA-3’	5’-GAGATTTGGAGCGGTGAGAA-3’	1.84
***MtRS2Z32* (*LOC112416407*)**	5’-GAGTGAAGAGCAGAAGCCC-3’	5’-GGTGATGGAGAGCGACG-3’	1.78
***MtTRXm4* (*Medtr2g079420*)**	5’-CTTCTCCCTTATTCGCTCTCC-3’	5’-CTCACACACGACGGTTCC-3’	1.71
***MtABA2* (*Medtr3g020670*)**	5’-GCTGTGGTAACTGGTGGAG-3’	5’-GGTCGTCTTGGATGTCGG-3’	1.74
***MtAQR* (*Medtr1g038815.1*)**	5’-GCCGAGAATAAAGAAAAGCCAAC-3’	5’-ATGACGAAAGCACCCCAAAG-3’	1.8
***MtAAG* (*Medtr7g093540*)**	5’CCCAAAACCACAACGCTTCA-3’	5’-CAAACGAGGGGCAAGGTCTA -3’	1.73
***MtEndoV* (*Medtr1g010130*)**	5’-CTTTGACAGGTTGCTCTGGG-3’	5’- TTGGCTCAGGGACACGATAC-3’	1.75
***MtPCNA* (*Medtr3g462130*)**	5’- GCAAAACACAAAGGCGGGA-3’	5’-GGAAGAGGGTGAAGAGGGAG-3’	1.71

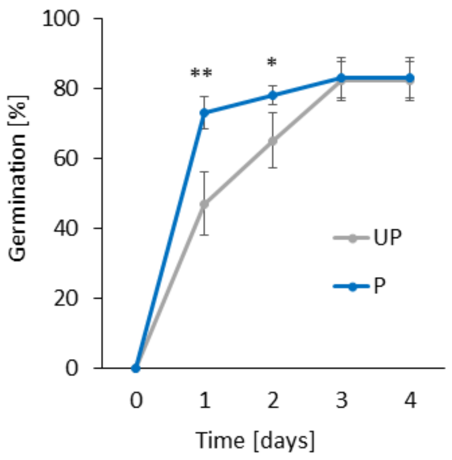

Fig. S1. Germination curve of *M. truncatula* unprimed (UP) and primed (P) seeds. Asterisks indicate statistically (heteroscedastic two-tailed Student’s *t*-test) significant differences; *, $P < 0.05$; **, $P < 0.01$; ***, $P < 0.001$.

Table S2. Seedling development parameters based on biometrical analyses carried out on *M. truncatula* four-days old seedlings developed from primed (P) and unprimed (UP) seeds. Asterisks indicate statistically significant differences determined using two-tailed heteroscedastic Student's *t*-test (*, $P < 0.05$; **, $P < 0.01$; ***, $P < 0.001$). RL, radicle length. FW, fresh weight. DW, dry weight.

Treatment	RL (mm)	FW (mg)	DW (mg)
UP	31.90 ± 5.95	39.53 ± 3.33	3.27 ± 0.43
P	33.40 ± 2.37	41.47 ± 5.07	2.93 ± 0.28

ROS detection

ROS (reactive oxygen species) levels were quantified in *M. truncatula* dry and imbibed seeds collected at the indicated timepoints, using the fluorogenic dye 2',7'-dichlorofluorescein diacetate (DCFH-DA; Sigma-Aldrich, Milan Italy). The dye is converted to a non-fluorescent molecule following deacetylation mediated by cellular esterases, and it is subsequently oxidized by ROS into the fluorescent compund 2',7'-dichlorofluorescein. DFC can be detected by fluorescence spectroscopy with maximum excitation and emission spectra of 495 nm and 529 nm, respectively. The assay was performed as described by Doria et al. (2019). *M. truncatula* seeds were collected at the indicated timepoints and dried on filter paper. Samples (three seeds per timepoint) were incubated for 15 min with 50 µl of 10 µM DCFH-DA and subsequently fluorescence at 517 nm was determined using a Rotor-Gene 6000 PCR apparatus (Corbett Robotics, Brisbane, Australia), setting the program for one cycle of 30 s at 25°C. As negative control, a sample containing only DCFH-DA was used to subtract the baseline fluorescence. Relative fluorescence was calculated by normalizing samples to controls and expressed as Relative Fluorescence Units (R.F.U.).

Reference

Doria, E., Pagano, A., Ferreri, C., Larocca, A.V., Macovei, A., Araújo, S., Balestrazzi, A. (2019) How does the seed pre-germinative metabolism fight against imbibition damage? Emerging roles of fatty acid cohort and antioxidant defence. *Front. Plant Sci.* 10:1-13. doi: 10.3389/fpls.2019.01505

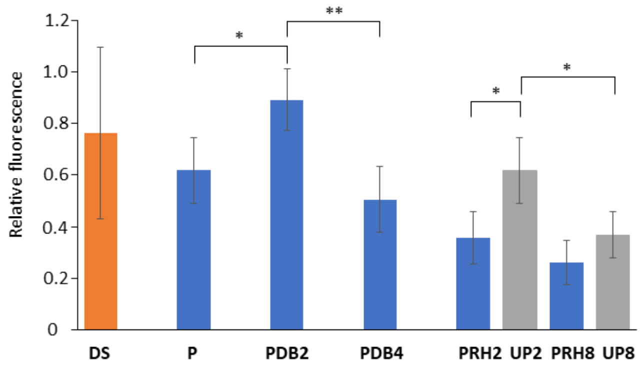

Fig. S2. ROS accumulation assessed through DCFH-DA Assay. DS, dry seed. P, hydropriming (2 h). PDB2 and PDB4, hydropriming followed by 2 h and 4 h of dry-back, respectively. PRH2 and PRH8, primed seeds after 2 h and 8 h of rehydration, respectively (post-priming imbibition). UP, unprimed. UP2 and UP8, unprimed seeds after 2 h and 8 h of rehydration, respectively (imbibition). Asterisks indicate statistically (heteroscedastic two-tailed Student's *t*-test) significant differences; *, $P < 0.05$; **, $P < 0.01$; ***, $P < 0.001$.

GO terms enriched in the identified proteins

The AgriGO software identified and classified 577 GO terms enriched in the identified proteins. The 24 GO terms listed within the first hierarchical level are shown in (Supplementary Fig. S3). Three main categories were represented: 'Biological Processes', 'Molecular Function', and 'Cellular Component'. Overall, the GO terms enriched in the Biological Processes in the *M. truncatula* experimental system hereby analysed were included in 11 main categories. The most enriched Go terms in Biological Processes were metabolic process (GO:0008152) and cellular process (GO:0009987). The enriched GO term in Molecular Function belonged to eight main categories. The most represented were binding (GO:0005488) and catalytic activity (GO:0003824). The enriched GO term in Cellular Component in the *M. truncatula* seed proteome belonged to the five main categories. The most represented were cell (GO:0005623) and cell part (GO:0044464).

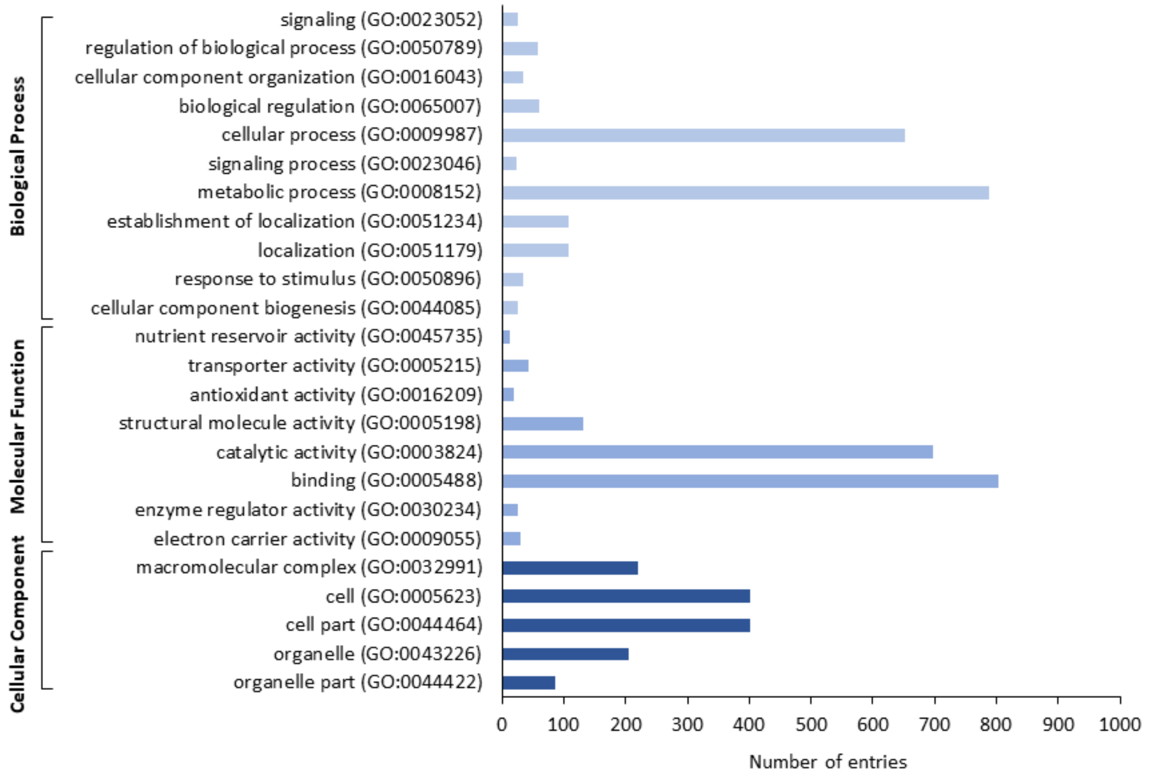

Fig. S3. Gene Ontology (GO) enrichment analysis of the *Medicago truncatula* protein entries identified. GO terms are listed as provided by the AgriGO software, within the first hierarchical level of the Biological Process, Molecular Function and Cellular Component.

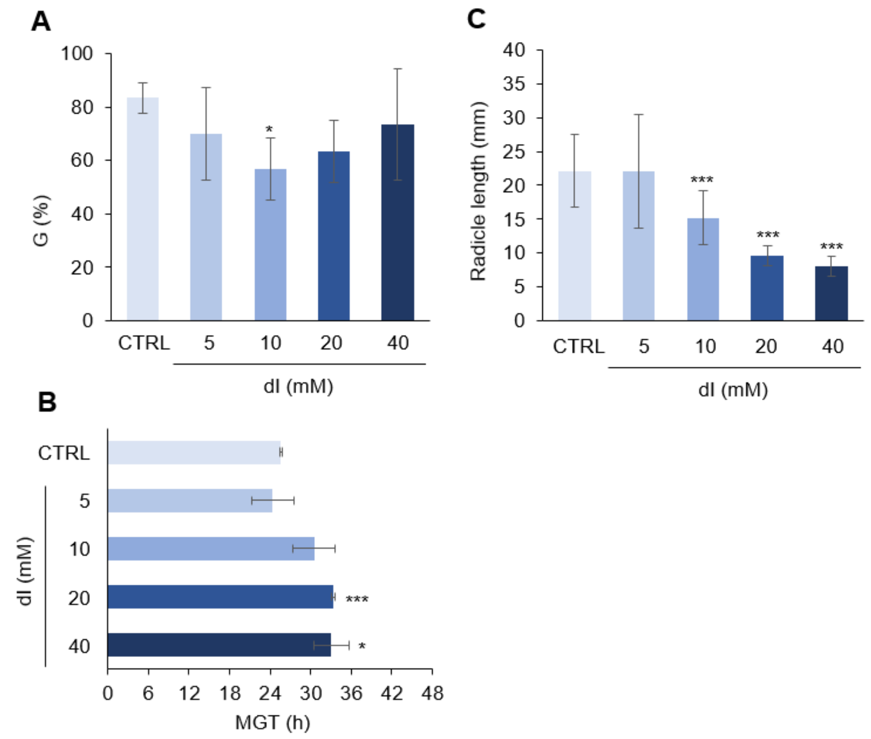

Fig. S4. Impact of increasing dI concentrations on *M. truncatula* seed germination performance and seedling development. **A.** Germinability (G). **B.** Mean Germination Time (MGT). **C.** Radicle length. dI, deoxy-inosine. Asterisks indicate statistically significant differences as determined through two-tailed Student's *t*-test. As for gene expression analysis *, $P < 0.05$; **, $P < 0.01$; *** $P < 0.001$.

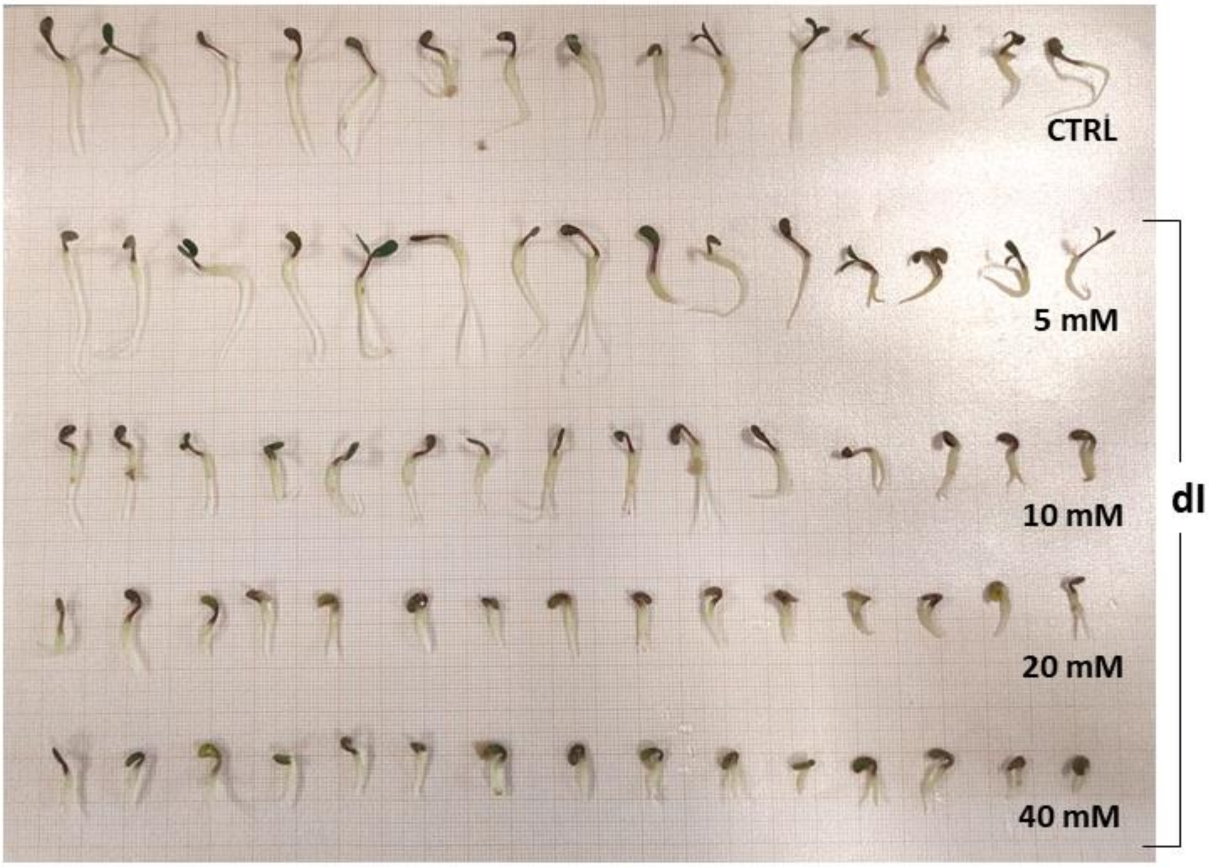

Fig. S5. Impact of increasing deoxy-inosine (dI) doses on the growth and phenotype of four-days old *M. truncatula* seedlings.

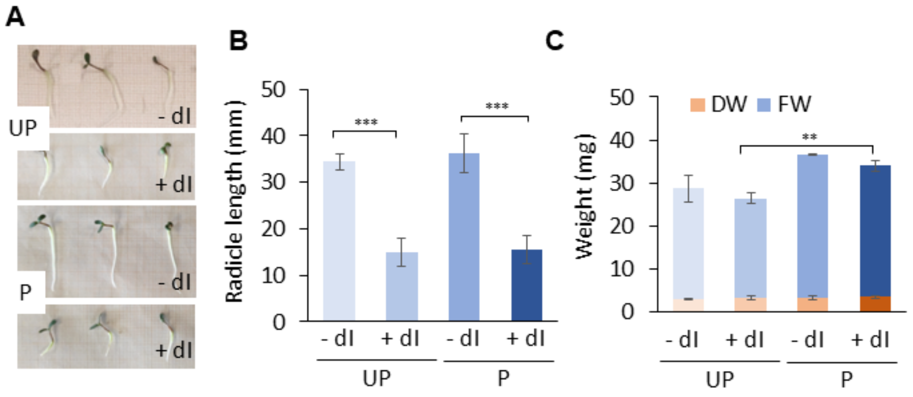

Fig. S6. Phenotyping and biometrical analyses. **A.** four-days old *M. truncatula* seedlings developed from untreated and primed seeds in presence/absence of 20 mM dI. **B**. Radicle length. **C.** Biomass measured as fresh weight (FW) and dry weight (DW), respectively. UP, unprimed. P, hydroprimed. DB, dry-back. - dI, absence of deoxy-inosine. + dI, 20 mM deoxy-inosine. Asterisks indicate statistically significant differences as determined through two-tailed Student's *t*-test. As for gene expression analysis *, *P* < 0.05; **, *P* < 0.01; *** *P* < 0.001.

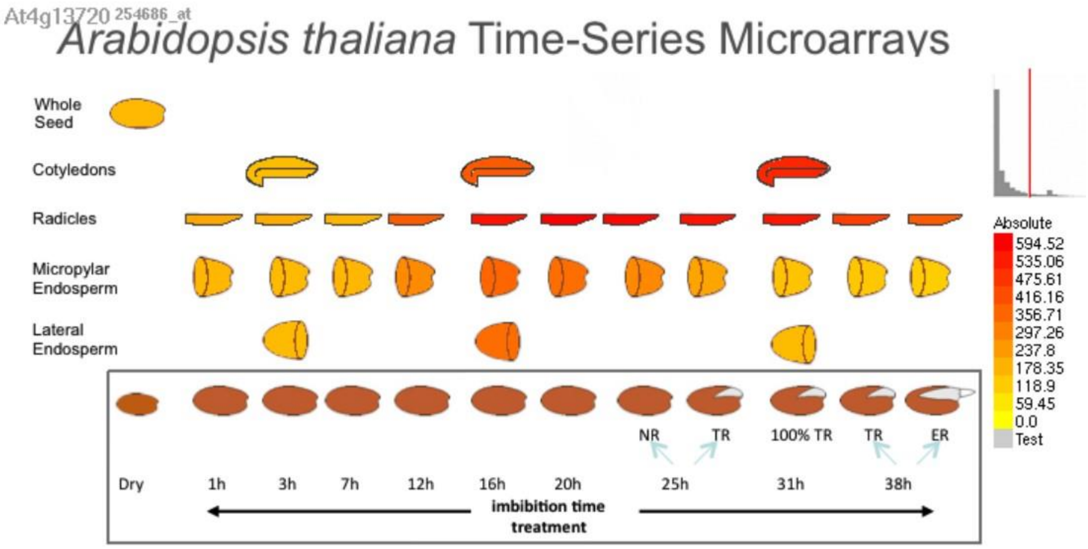

Fig. S7. Levels of *AtITPA* transcript detected in *Arabidopsis thaliana* seeds during germination. The expression profiles of the *AtITPA* gene (accession N° At4g13720) were retrieved using the the online network query tool Nottingham Seed eFP browser (http://ssbvseed01.nottingham.ac.uk/efp_browser/efpWeb.cgi) which is part of the Virtual Seed Web Resource (vseed.nottingham.ac.uk) (Dekker et al., 2013).

Reference

Dekkers, B.J.W., Pearce, S., van Bolderen-Veldkamp, R.P.,sMarshall, A., Widera, P., Gilbert, J., Drost, H-G., Bassel, G.W., Müller, K., King, J.R., Wood, A.T.A., Grosse, I., Quint, M., Krasnogor, N., Leubner-Metzger, G., Holdsworth, M.J., Bentsink, L. (2013) Transcriptional dynamics of two seed compartments with opposing roles in Arabidopsis Seed germination. *Plant Physiol.* 163: 205-215. doi: 10.1104/pp.113.223511

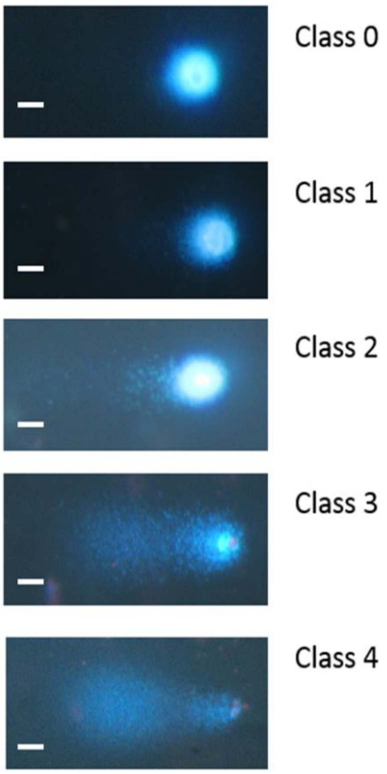

Fig. S8. Representative pictures of the morphological classes of nuclei observed in alkaline single cell gel electrophoresis (Comet Assay). Scale bar 50 µm.
